# Solvent-Site Prediction for Fragment Docking and Its Implication on Fragment-Based Drug Discovery

**DOI:** 10.1021/acs.jcim.5c02352

**Published:** 2025-11-24

**Authors:** Laura Almena Rodriguez, Vera A. Spanke, Christian Kersten

**Affiliations:** † Institute of Pharmaceutical and Biomedical Sciences, 9182 Johannes Gutenberg-University Mainz, Staudingerweg 5, Mainz 55128, Germany; ‡ Department of General, Inorganic and Theoretical Chemistry, and Center for Molecular Biosciences Innsbruck, University of Innsbruck, Innsbruck 6020, Austria; § Institute for Quantitative and Computational Biosciences, Johannes Gutenberg-University, BioZentrum I, Hanns-Dieter-Hüsch Weg 15, Mainz 55128, Germany

## Abstract

The accuracy in the posing and scoring of low-affinity fragments is still a main challenge in fragment-based virtual screenings. The positive impact of including structural or predicted water molecules during docking on the docking performance is discussed frequently and is not conclusive so far. We present a comprehensive statistical evaluation of the effect of including crystallographic or predicted water molecules on the docking performance of fragment redocking. Further, cross-docking fragments into binding sites occupied by larger ligands and *vice versa* were elucidated. These cross-dockings imitate realistic use cases of fragment hit identification and fragment growing or synthon-based virtual screenings, respectively. Therefore, a new benchmark data set, called Frag2Lead containing 103 fragment-protein and corresponding lead-protein complexes, was compiled. Inclusion of water molecules during docking had a general positive impact on docking performance, but the preferred combination of the docking tool and water model varied across the different targets. A consensus approach over multiple solvent models and docking tools turned out to be beneficial for both re- and cross-dockings. Implementing constraints by template docking or pharmacophore features is advantageous for pose prediction for fragment growing approaches.

## Graphical abstract

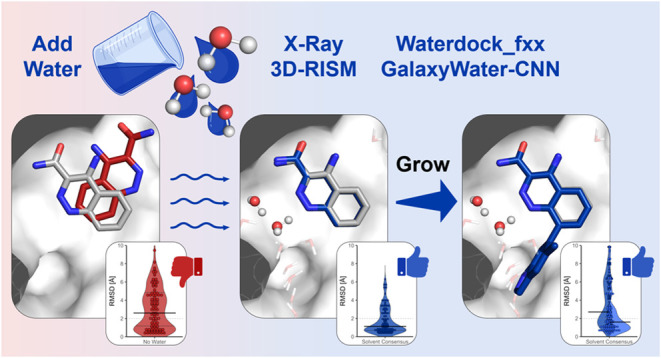

## Introduction

### Fragment-Based Drug Discovery (FBDD)

Traditionally, many hits are identified via high-throughput screening (HTS). Hits emerging from HTS usually show moderate affinities in the low-μM to nM range and molecular weights above 250 Da. In contrast, FBDD approaches represent an alternative to HTS with several advantages. Fragments are low-molecular-weight (<300 Da), low-complexity molecules. Thus, a (typically 1000 times) smaller library for fragment-based screening (FBS) covers chemical space more efficiently.^1^ Due to lower complexity, fragments have a higher probability of matching the binding site.^2^ The resulting fragment hits usually bind with low affinities,^1^ but a high proportion of fragment atoms is involved in few high-quality interactions with the target site, which makes them more efficient binders.^3^ This is usually described by a higher ligand efficiency (LE)^4^ compared to HTS hits. Starting the hit optimization from an efficient fragment will more likely lead to a potent lead with acceptable physicochemical properties.^5^ After fragment hit identification, the potency and molecular properties are usually optimized by rational, structure-based drug design (SBDD) using fragment growing, merging, or linking. The interplay between structural and computational methods, such as X-ray crystallography and molecular docking, is often fundamental to guide SBDD.^6^ FBDD was already implemented successfully in several drug discovery campaigns including hard-to-drug targets like B-cell lymphoma (BCL)-2.^7^ Over 50 FBDD-derived compounds have entered clinical development so far. With the approval of capivasertib at the end of 2023, there are now eight U.S. Food and Drug Administration (FDA)-approved compounds derived from fragments.^8^ In 2022, 18 ^9^ and in 2023, 17 ^10^ successful fragment-to-lead studies were published, which include the development of three clinical candidates.^9^

### Fragment Docking and Synthon-Based Virtual Screening (VS)

VS of commercially available compound libraries has been regarded as a scope-widening, time- and cost-saving alternative to physical HTS and FBS in hit identification.^11^ The accuracy of docking and scoring fragments is still a main challenge in fragment-based VS.^12,13^ Due to their small size, fragments can be placed in energetically closely related minima poses, resulting in incorrectly predicted binding modes compared to the crystallographic binding mode. In addition, fragments are weak binders, showing a few interactions with their target and thus weak binding free energies. Most of the scoring functions used for drug-like^14^ compounds cannot differentiate sufficiently between weak binders and nonbinders.^12,13,15^ As the library size increases, postprocessing of the VS results and selection of potential candidates become more important.^15^ Various methods including different types of scoring functions were used in FBDD.^12,16,17^ Exemplarily, the docking programs DOCK,^16^ GOLD,^18^ and SEED^19^ combined with different rescoring functions have been demonstrated to be suitable to prioritize fragments for further optimization in case studies.^17,19–23^ In 2020, statistical analysis of different combinations of docking programs and rescoring functions emphasized the benefit of applying additional rescoring functions for fragment docking.^24^ In addition, pharmacophore constraints can be applied in fragment-based VS campaigns^3,25^ and implementation of multiple short molecular dynamic simulations increased fragment posing accuracy.^26^

In the last years, virtual on-demand libraries emerged.^27^ These libraries can be maintained as nonenumerated chemical spaces to facilitate the handling of ultralarge virtual libraries. Chemical spaces are combinatorial fragment spaces and defined by the combination of their building blocks, so-called synthons, and their corresponding reaction pathways.^28^ Common chemical spaces are Enamine’s REAL Space comprising 76.9 billion^29^ or OTAVA’s CHEMriya Space with 55 billion^30^ make-on-demand compounds among others. Pharmaceutical companies have created proprietary spaces like GlaxoSmithKline with GSK XXL space^31^ having the largest described chemical space so far featuring 10^26^ compounds.^32–34^ While the increasing size of chemical spaces does not necessarily increase diversity,^35^ the overlap between commercially available spaces is surprisingly low.^28^ Thus, molecular docking using chemical spaces or VS in general can lead to more novel and diverse hits in comparison to VS of only in-stock compounds.^28,36^ Since an exhaustive molecular docking screening, referred to as brute-force docking, is not feasible at this scale, approaches to reduce the computational time and cost have been made. Machine learning models were integrated in the docking workflow, yielding high hit rates and manifold reduction in computing costs.^37–40^ Selecting a diverse subset of the library by clustering can lead to the undesired discard of unpredictable active molecules within a cluster.^41,42^ In contrast, the design of a target-specific focused virtual library based on known binding scaffolds was beneficial, showing high hit rates.^43,44^ In addition, crystallographic binding poses of known binders can be implemented in template-guided docking procedures and were shown to be a useful starting point in FBDD.^45,46^ Docking of nonenumerated chemical spaces and thus docking of the respective synthons (fragments) is an efficient way to circumvent resource-intensive brute-force docking of the fully enumerated library. After initial synthon docking, synthon hits can be enumerated through the corresponding reaction pathways for extension. These resulting sublibraries of drug-like molecules can then be docked. Synthon-based docking was applied successfully in several studies.^25,46–49^ A combination of chemical space docking and crystallographic hit discovery demonstrated high screening success rates of around 40% and the whole process could be accomplished in only 9 weeks.^46^ The initial fragment-like synthon docking within such a synthon-based virtual screening has to be accurate to ensure valuable starting points for enumeration.

### Hydration Sites

Besides accurate scoring functions and addressing target’s flexibility, one of the main challenges in molecular docking is the displacement of or interaction with, and position prediction of structural water molecules within target-binding sites.^50,51^ Water molecules located at target-ligand interfaces can be involved as hydrogen bond donors or acceptors in interactions with other water molecules, the ligand, the target, or mediating ligand-target interactions.^52–54^ Analysis of 19 high-resolution crystal structures of protein–ligand complexes emphasized that most of the water molecules (nearly 80%) within binding sites are involved in bridging interactions with three or more hydrogen bonds stabilizing protein–ligand interactions.^55^ Due to their interactions, water molecules located in binding sites are typically more restricted in their translation and rotation compared to bulk water.^56^ Release of a restricted water molecule upon ligand binding increases entropy, which can contribute positively to the binding affinity of the ligand.^52,57–59^ But a missing recovery of the enthalpic interactions made by the water molecule with the target after water displacement can result in a decrease in affinity. In such cases, water molecules should not be displaced and rather considered as part of the binding site.^52,58–60^ Water molecules can also play a critical role in selectivity when different hydration patterns are present in proteins with conserved binding sites.^61,62^ Experimentally, X-ray crystallography is the most common method to identify water molecules. Still, accurate placement of water molecules within the electron density map can be difficult, even if the resolution of the crystal structure is under 2 Å. Moreover, hydrogen atoms are not resolved and the orientation of the water molecules cannot be measured directly.^63^ Besides the position, the thermodynamic profile of water molecules is not easily accessible experimentally or computationally due to overlaying effects.^58^

### Solvent Prediction and Docking

Several computational methods have been developed to overcome the experimentally limited possibilities of identifying structural water molecules. Knowledge-based site prediction tools use similar crystallographic data to determine water sites^64^ and can be coupled to deep-learning approaches.^65^ Interaction-based site predictions determine which sites are more favorable for the water molecule by sampling possible locations via grid-based probe and flooding-based sampling methods.^64^ The free energy methods estimate the free energy of binding for water molecules often with high accuracy and are usually obtained from time-consuming molecular dynamics simulations.^64,66,67^ A study with four water prediction tools and three lead-protein complexes revealed that including the predicted water sites is usually helpful to address lead optimization problems and to improve docking predictions.^68^ Including crystallographic or simulated water molecules during the docking process is handled differently across different docking programs, and the effects of the included water molecules on the docking performance are discussed. In some studies, inclusion of specific water molecules did not significantly improve pose prediction accuracy. Ligands displacing these water molecules could not be docked at all.^69^ An approach to score water mediation and displacement within a docking process revealed only small improvements for water-mediated complexes.^50^ Contrarily, case studies^70–74^ and statistical studies^75–78^ demonstrated that including structural water molecules or water models improves docking predictions. It is recommended to limit the number of water molecules to those that are known to be crucial for ligand binding.^50,71,72^ Moreover, the implementation of structural water molecules as part of the receptor in a VS approach can lead to unique hits.^70^ A cross-docking study with six different proteins (each having crystal structures with several different ligands) showed that the inclusion of conserved water molecules increases cross-docking accuracy in a case-dependent manner. Nevertheless, the authors recommend including water molecules whenever possible.^73^ Evaluation of combinations of three docking programs and including no water, crystallographic, and predicted water molecules on two systems showed an improvement in redocking root-mean-square deviation (RMSD) for solvated systems.^79^

Taken together, inclusion of water molecules can have a positive impact on docking performance, but often is dependent on the used targets and docking programs and thus not generally conclusive.^51^ Especially FBDD, VS, and fragment-to-lead optimizations can benefit from an accurate pose prediction. Likewise, synthon-based ultralarge library dockings rely on accurate predictions of the initial fragment-sized synthons. We present a comprehensive statistical evaluation of the effect of including crystallographic water molecules as well as water prediction models on the docking performance of fragment redocking. In addition, water’s influence on cross-docking fragments into binding sites occupied by larger ligands and *vice versa*, imitating realistic use cases of fragment docking for hit identification or synthon docking, and fragment growing was evaluated, respectively. We used two different data sets comprising around 100 protein-fragment complexes each. The three docking setups of FlexX,^80^ FlexX with HYDE rescoring,^81,82^ and DOCK^16^ were combined with target preparation excluding solvent, using crystallographic water molecules and predicted solvent sites by 3D-*RISM*,^83^ WaterDock,^84,85^ Galaxywater-CNN,^65^ and an in-house method of docking water molecules with FlexX (in the following referred to as waterdock_fxx, Supporting Information).

## Materials and Methods

### Data Set Preparation

The impact of solvent models on fragment redocking was evaluated using the LEADS-FRAG data set. LEADS-FRAG contains 93 high-quality protein-fragment complexes.^24^ For evaluation of cross-dockings, a new data set was compiled from Fragment-to-Lead campaigns containing protein-fragment and protein-lead complexes.^86–91^ The paper series *Fragment-to-Lead Medicinal Chemistry Publications* ^86–91^ was selected for this purpose, which gave a tabulated overview of successful Fragment-to-Lead campaigns listing the corresponding Protein Data Bank (PDB) entries of target-fragment and target-lead complexes, if available (Table S10, Figure S13). Structures were aligned using PyMOL (PyMOL 2.4.1, Open-Source Build, Schrödinger, LLC, http://www.pymol.org) and further processed. All structures were visually inspected and corrected using MOE (Molecular Operating Environment (MOE); 2020.09; Chemical Computing Group ULC: 1010 Sherbrooke St. West, Suite #910, Montreal, QC, Canada, H3A 2R7, 2020, https://www.chemcomp.com/index.htm) if necessary prior to molecular docking. The resulting data set (in the following referred to *Frag2Lead*) contains 103 fragment-protein and corresponding aligned lead-protein complexes, which are provided in the Supporting Information.

### Docking Programs

Three docking setups were used to evaluate the influence of the water models on redocking and cross-docking. FlexX^80^ (FlexX Version 5.1.0, BioSolveIT GmbH, St. Augustin, Germany, https://www.biosolveit.de/FlexX) with and without HYDE^81,82^ rescoring (hydescorer version 1.4.0, BioSolveIT GmbH, St. Augustin, Germany, https://www.biosolveit.de/HYDE) as well as DOCK (DOCK^16^ version 6.9,^92^ University of California, San Francisco, 2018, https://dock.compbio.ucsf.edu) was used. All dockings were performed using an automated workflow with minimal user intervention. FlexX/HYDE was selected for docking due to its simple automated solvent handling. In the initial pose generation, FlexX considers water molecules with at least three potential interactions with the binding site; all other binding site water molecules can be displaced during docking. In HYDE rescoring, water molecules forming at least three hydrogen bond interactions either to the binding site or the ligand are considered to refine and rescore the poses. This simplifies water molecule selection for docking compared to setting cut-offs based on predicted water energies. In contrast, DOCK6.9 has no preselection of water molecules for docking. Keeping all water molecules in a defined orientation from crystal structures or prediction (in the following referred to as DOCK) might introduce too much bias toward the reference complex. This is especially problematic for cross-docking with a larger ligand than the reference ligand, as the binding site might become too small. Therefore, in a modified docking protocol, only water molecules from HYDE optimization were kept for docking with DOCK (in the following referred to as DOCK_mod). In this case, the H-bond network optimization feature of HYDE was used to identify water molecules that form three interactions with binding site objects by using the experimental binding mode with crystallographic or predicted water molecules.

#### FlexX

Docking was performed using extracted ligands in the sd-file format. Ligand protonation and tautomerization are automatically performed within FlexX by Protoss.^93^ 20 poses per ligand were generated. For FlexX, only the top pose was considered for the RMSD calculation. Flip stereo mode was turned on for decoys with an undefined stereochemistry.

#### HYDE

Top 20 poses from FlexX docking were rescored using HYDE. Poses were re-sorted by HYDE-score, and the top pose was used for RMSD calculation. This procedure is in the following referred to as HYDE.

#### DOCK

The ligands were protonated, and AM1-BCC^94^ charges were assigned using OpenEye Applications (2023.1.0) QUACPAC molcharge (QUACPAC 2.2.2.0. OpenEye, Cadence Molecular Sciences, Santa Fe, NM. http://www.eyesopen.com). Cofactors were treated equally. The receptors were prepared using MOE. All organic molecules that were not part of the binding site were removed. For parameters for sphere, box, and grid generation as well as for flexible ligand docking, default values were used based on the DOCK6 tutorial.^95^

#### DOCK_mod

For receptor preparation, the H-bond network optimization feature of HYDE was applied on the complex structures with crystallographic or predicted solvent. The receptor was further prepared and docked as described for DOCK.

#### Pharmacophore (ph4) Constrained Docking

The pharmacophore features were created in SeeSAR (SeeSAR Version 11.2.2, BioSolveIT GmbH, St. Augustin, Germany, https://www.biosolveit.de/SeeSAR) and used for FlexX and HYDE lead-in-fragment cross-docking. One pharmacophore feature per fragment (Frag2Lead data set) was created based on the highest HYDE-score contribution per atom with a pharmacophore radius of 1.5 Å. The docking definition was exported from SeeSAR for FlexX and HYDE docking.

#### RMSD Calculation

All RMSD values were calculated using obrms from Open Babel^96^ (Open Babel version 3.1.1, 2021, http://openbabel.org), which corrects for molecular symmetry.^97^ For statistical analysis, RMSD values of ≤2.0 Å are considered as a successful pose prediction.

Figures were made with PyMOL and plots with RStudio (RStudio 2023.12.1 + 402 “Ocean Storm”, Posit team (2024). RStudio: Integrated Development Environment for R, Posit Software, PBC, Boston, MA. URL http://www.posit.co/).

### Solvent Models

Different solvent models were elucidated: no water referred to as *dry*, crystallographic water referred to as *wet*, predicted water molecules by 3D-*RISM* ^98^ generated within MOE referred to as *rism*, predicted water sites by WaterDock2.0 ^84^ referred to as *waterdock*, predicted water sites by docking water molecules using FlexX referred to as *waterdock_fxx* (see Supporting Information for scripts and methodological details) and predicted water sites by Galaxywater-CNN^65^ referred to as *gw*. The SPAM^99^ method was tested for selected entries in the LEADS-FRAGS data set for comparison as a computationally more expensive molecular dynamics (MD) simulation technique.

#### Wet

The receptors were not modified after data set generation.

#### Dry

All water molecules of the *wet* receptors were removed by using PyMOL.

#### Rism

3D-*RISM* (three-dimensional reference interaction site model) was developed in 1997 and is a statistical mechanics method.^83^ Statistical mechanics methods consider target flexibility and fluctuations of water molecules in a statistical manner. 3D-*RISM* takes solute–solvent electrostatic and van der Waals interactions as well as solvent packing into account. An advantage of this method is that it can represent water molecules as nonspherical, oriented molecules. Thus, hydrogen bond networks formed by water molecules are modeled more efficiently and in a faster way. The 3D-*RISM* method used is implemented in MOE. To create the *rism* receptor, the *dry* receptor was first prepared via Quickprep to correct missing atoms and protonate the complex structure.^100^ Quickprep was performed without energy minimization to keep atom coordinates comparable to those of the other docking and solvation methods. Solvent analysis was performed for the binding site (7 Å around the ligand), and water molecules were subsequently extracted to build the solvated complex for docking.

#### Waterdock

The WaterDock^84,85^ method belongs to the interaction-based site predictions and uses AutoDock Vina^101^ to repeatedly dock water molecules into the binding site. After docking, the water molecules are filtered according to their docking score, and the remaining water molecules are clustered into discrete water sites.^84,85^ This approach considers one water molecule at a time. Hence, water sites are missing, where hydrogen bonds between two water molecules would have been important.^64^ For WaterDock, protonated ligands were converted to pdb file format using Open Babel and prepared receptors were converted into pdbqt file format using AutoDockTools^102^ within MGLTools (MGLTools version 1.5.6, Molecular Graphics Laboratory (MGL), https://ccsb.scripps.edu/mgltools). The command line version of WaterDock2.0 was used for docking (https://github.com/bigginlab/WaterDock-2.0).^84^

#### Waterdock_fxx

This self-made solvent model is similar to WaterDock, but the docking software to repeatedly dock water molecules is FlexX and no initial docking score cutoff and hydrogen bond saturation limit for functional groups were implemented. This method can be used with either a clustering approach or a cascade method to select specific water molecules. The cascade method selects the best-scoring water molecule, deletes all neighboring water molecules within the adjustable method distance, and chooses the next best water molecule from the remaining ones. The clustering method selects the best-scoring water molecule in each cluster. Apo and holo structures can be used, which must be specified in the input. The minimal distance between water molecules and the ligand can be adjusted for holo structures. Scripts (flexx-waterdock.py for docking a water molecule into the binding site and topwater.py for solvent-site selection) to use with FlexX are provided in the Supporting Information. In this study, the holo cascade approach was used with a minimal distance to the ligand of 3.0 Å and minimal distance between water molecules of 2.8 Å (cascade method). Further details are provided in the Supporting Information under the section Extended Material and Methods–Implementation of *waterdock_fxx*, Figure S14.

#### GW

GalaxyWater-CNN^65^ predicts hydration sites from a 3D-convolutional neural network (CNN) model generating a water score map on a particular protein structure of a protein or protein–ligand complex. It predicts only the hydration site without extracting thermodynamic information. The *dry* receptor–ligand complex was used as the input structure. Three different hydrated output structures can be exported depending on the score cutoff for water molecules (34, 38, and 42 with 34 having the most water molecules). For the first data set LEADS-FRAG, all score cut-offs were implemented in docking studies. In the Frag2Lead data set, only receptors with a score cutoff of 34 were implemented.

#### SPAM

SPAM^99^ (maps spelled backward) belongs to the statistical mechanics-based methods and uses results from explicit solvent MD simulations. In MD, the molecular motion, in this case including explicit solvent, as a function of time (and temperature) is simulated. SPAM evaluates the distributions of interaction energies in the solvated complex to calculate the enthalpy and entropy for discrete water molecules. SPAM was shown to identify hydration sites and to provide additional thermodynamic information.^58,99^ The SPAM method was implemented for selected entries from the LEADS-FRAG data set. For MD simulations, the receptors were prepared with the MOE Quickprep functionality without energy minimization. Missing loops were corrected via the MOE loop modeler. Parametrization of the ligands and the cofactors was done using AmberTools22 ^103^ with antechamber^104,105^ 22.0 using AM1-BCC charges^94^ and gaff2 atom types.^105^ The module tLEaP^103^ was used to prepare the receptor and complex. For the receptor, the Amber ff14SB^106^ force field set was applied. The complex was minimized using implicit solvent with sander.^103^ The system was solvated with TIP3P water^107^ and neutralized by the addition of sodium or chloride ions. The system was placed in a periodic boundary box with a minimum distance of 10.0 Å between the protein and box walls. Before MD simulation, the system was relaxed by 10,000 steps of energy minimization followed by a 1 ns heating protocol with gradually decreasing harmonic constraints on protein and ligand atoms. 10 ns long MD simulations were performed for the respective entry with the time step set to 2.0 fs and fixed bond lengths. The temperature was held constant at 300 K. The MD equilibrations and productive runs were performed using NAMD^108^ 2.14 (http://www.ks.uiuc.edu/Research/namd/). The conserved hydration sites were evaluated and their coordinates exported using AMBER’s trajectory analysis and manipulation tool CPPTRAJ^109^ version V6.18.1 from AmberTools22. Mapping oxygen atoms of water molecules by volumetric map calculation (volmap) was adjusted to 0.5 Å grid spacing, a scaling factor applied to atom radii 1.36, the size of the volumetric box set to 8 Å, and the threshold for peak detection set to 0.07. The SPAM module of CPPTRAJ was used to analyze the generated solvent density peaks. Water molecule densities are clustered with a distance cutoff of 12 Å and a cubic volume with an edge length of 2.5 Å. Bulk solvation free energy per mole of solvent was adjusted to −28.0811 kcal/mol, and the bulk enthalpy of solvation was adjusted to −19.844 kcal/mol (references for TIP3P generated from a pure 40 × 40 × 40 Å solvent box simulation of 10 ns). The calculated hydration sites were converted to pdb format by Open Babel and added to the *dry* receptor by PyMOL to generate the *spam* receptor for docking.

### Binder-Decoy Discrimination

In the described data sets, only the crystallographic reference ligands are considered binders. The F2X-Entry library^110^ containing 96 structurally diverse fragments was used as decoys. Molecular descriptors for the F2X-Entry data set, the LEADS-FRAG data set, and the fragments of the Frag2Lead data set were calculated using MOE (Table S8 and Figure S13). 3D conformers of all fragments were generated with OMEGA^111^ (OMEGA 4.2.1.1 OpenEye, Cadence Molecular Sciences, Santa Fe, NM. http://www.eyesopen.com.). The docking scores of the F2X fragments for each target were compared to the score of the respective ligand for ranking.

## Results

### LEADS-FRAG Data Set Redocking

Fragment redocking accuracy in dependence of crystallographic or predicted solvent molecules was evaluated using 93 protein-fragment complexes from the LEADS-FRAG data set.^24^ This data set contains high-resolution (<2.0 Å), high-quality (*R*_free_ > 0.3) protein-fragment (100–300 Da) complexes. Docking was performed without water molecules (*dry*), crystallographic water molecules (*wet*), and predicted water molecules using 3D-*RISM* (*rism*), WaterDock2.0 (*waterdock*), docking-placed water molecules using FlexX (*waterdock_fxx*), and Galaxywater-CNN (*gw*), respectively. Dockings were conducted using FlexX with and without HYDE rescoring and DOCK, as well as DOCK with water molecules preselected by HYDE based on their interaction capacities with the binding site residues and the reference ligand (referred to as DOCK_mod). For Galaxywater-CNN, different score cut-offs for water molecules were tested, which are referred to as *gw34*, *gw38*, and *gw42*. The score cutoff had only minor effects on the docking performance (Figure S1, Table S1). The receptor implying the highest number of water molecules (*gw34*) was used to proceed in this study. Further, from the methods placing water by docking, *waterdock* was found to be inferior to the in-house *waterdock_fxx* model in terms of success rates and technical issues requiring manual corrections (Table S2). Therefore, *waterdock* was not further followed up beyond the LEADS-FRAG data set redocking.

There were only minor statistical variations between different water models for FlexX in redocking success rates (defined as ligand RMSD ≤ 2.0 Å) between 53 and 58% (Table 1, Figure S2A, and Table S7). When HYDE rescoring was applied (Table 1 and Figure S2B), the success rates slightly decreased except for *rism* and *waterdock_fxx* (Table 1). As during HYDE refinement and rescoring, the same amount of water molecules or even more are considered than for FlexX scoring alone, it is reasonable that this method is more sensitive to solvent molecules in the binding site. In contrast, DOCK showed significant differences among the solvent models (Table 1 and Figure S2C). The *dry* model resulted in a success rate of only 35%, while the *wet* and the *gw* water model, in combination with DOCK, improved pose prediction to be correct in 59% and 60% of the cases, respectively. For *rism*, the redocking RMSD distribution showed two maxima with either very precise RMSDs (<2 Å) or diverging RMSD values (>10 Å) (Table 1 and Figure S2C). DOCK considers all explicit water molecules leaving nearly a template of the ligand’s shape (Figure 1 and Figure S4). When applying the filtering step for water molecules meeting HYDE water handling criteria (DOCK_mod), the docking performance decreased (Table 1 and Figure S2D) except for *rism* (45% success rate). Overall, FlexX redocking performed best, but was less sensitive to water models compared to HYDE rescoring and DOCK (Table 1). Previous studies showed that including all crystallographic water molecules increased redocking success rates but is not rational for drug discovery in contrast to only including key water molecules.^71,112^

**Figure 1:**
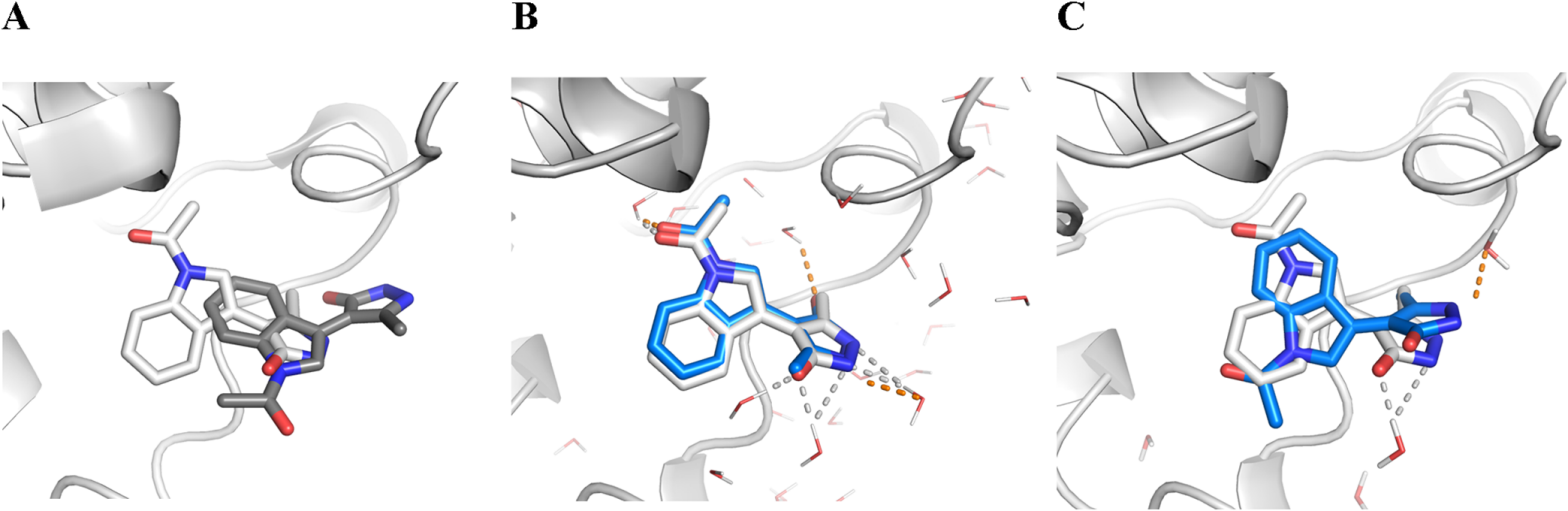
Redocking pose comparison of (A) DOCK *dry* (RMSD = 4.9 Å), (B) DOCK *wet* (RMSD = 1.8 Å), and (C) DOCK_mod *wet* (RMSD = 3.2 Å) for the BRPF1 bromodomain (PDB-ID: 5D7X^113^) from the LEADS-FRAG data set. The crystallographic reference ligand is shown in white, docking poses in gray for *dry* and blue for *wet*. Polar contacts of the docked ligand to water molecules are illustrated as orange dashed lines and in gray for interactions of the reference ligand.

**Table 1:** LEADS-FRAG Data Set Redocking Success Rates in % (Number of Data Points) Defined as Predicted Binding Modes with RMSD ≤ 2.0 Å Compared to Crystal Structure^a^

**software**	***dry***	***wet***	***rism***	***waterdock_fxx***	***gw34***
FlexX	54% (91)	58% (91)	54% (91)	57% (91)	53% (89)
HYDE	43% (91)	46% (91)	54% (91)	54% (91)	45% (89)
DOCK	35% (92)	59% (92)	45% (92)	43% (92)	60% (90)
DOCK_mod	35% (92)	41% (92)	46% (92)	39% (92)	42% (90)

a Visualization as violine plots is shown in Figure S2.

While for DOCK, addition of all water molecules from the crystal structure or prediction improved redocking, these solvent constraints limit space and interactions to similarly small molecules leaving no space for *in silico* fragment growing or merging (Figure 1B and Figure S4). The introduction of a less restricted water network with DOCK_mod was therefore necessary despite its worse redocking performance (Table 1, Figures S2C,D and S4). The receptors of DOCK_mod offered the possibility of growing from the initial fragment (Figures 1A–C and S4).

For 20 examples, the MD-based water model *SPAM* was evaluated (Tables S3 and S4). *SPAM* solvent models showed no general benefit with equal or inferior redocking performances compared to *dry*, *wet*, or *rism*. Thus, this computationally expensive, simulation-based method was not followed up.

### Binder-Decoy Discrimination

In addition to accurate pose prediction, molecular docking needs to discriminate binders from nonbinders by the scoring function in virtual screening applications. Ideally, known nonbinders from HTS or fragment screenings are used. However, such information is very heterogeneous due to different assays and hit selection criteria or not disclosed for screenings from the pharmaceutical industry. Therefore, usually property-matched decoys from known ligands are generated for this purpose. Decoys bear similar physical-chemical properties like their corresponding ligand,^114–117^ but have different topologies. With only one binder, the crystallographic reference ligand, discrimination from decoys by the receiver-operating characteristic (ROC) area under the curve (AUC) is difficult to interpret. However, crystallographic reference ligands are usually recovered within the top 5% of a data set containing one ligand per 100 decoys.^118^ Further, fragments only form few interactions with the target, and decoys containing the respective moiety from property matching may be able to mimic this interaction pattern, hence not well serving as a surrogate for potential nonbinders. To simplify the respective potential nonbinder surrogate data set and analysis, the 96 structurally diverse fragments of the F2X-Entry data set^110^ were used as decoys for all targets (Table S8). The probability that a random fragment is predicted to be a binder is expected to be smaller than that of a decoy with similar physical-chemical properties. A poor rank of the ligand despite good pose can arise from decoys scoring unexpectedly well due to their competitive appearance.^110,118^ The F2X fragments show overall similar physicochemical properties like the fragments from the LEADS-FRAG data set (Table S6). Rankings of the fragments within the F2X-decoys (Tables 2 and S9) by docking score showed that FlexX performed the best. In 75% (=third quartile) of the cases, the ligand appears in the top 27–32 of best scored molecules. Solvent models had minor effects on ranking as determined by FlexX. HYDE, DOCK, and DOCK_mod showed inferior results compared to FlexX. DOCK in combination with *wet* and *gw34* performed the best among HYDE, DOCK, and DOCK_mod (Table 2 and Figure 2). Similar trends were already observed for pose prediction in the redockings (Table 1).

**Figure 2:**
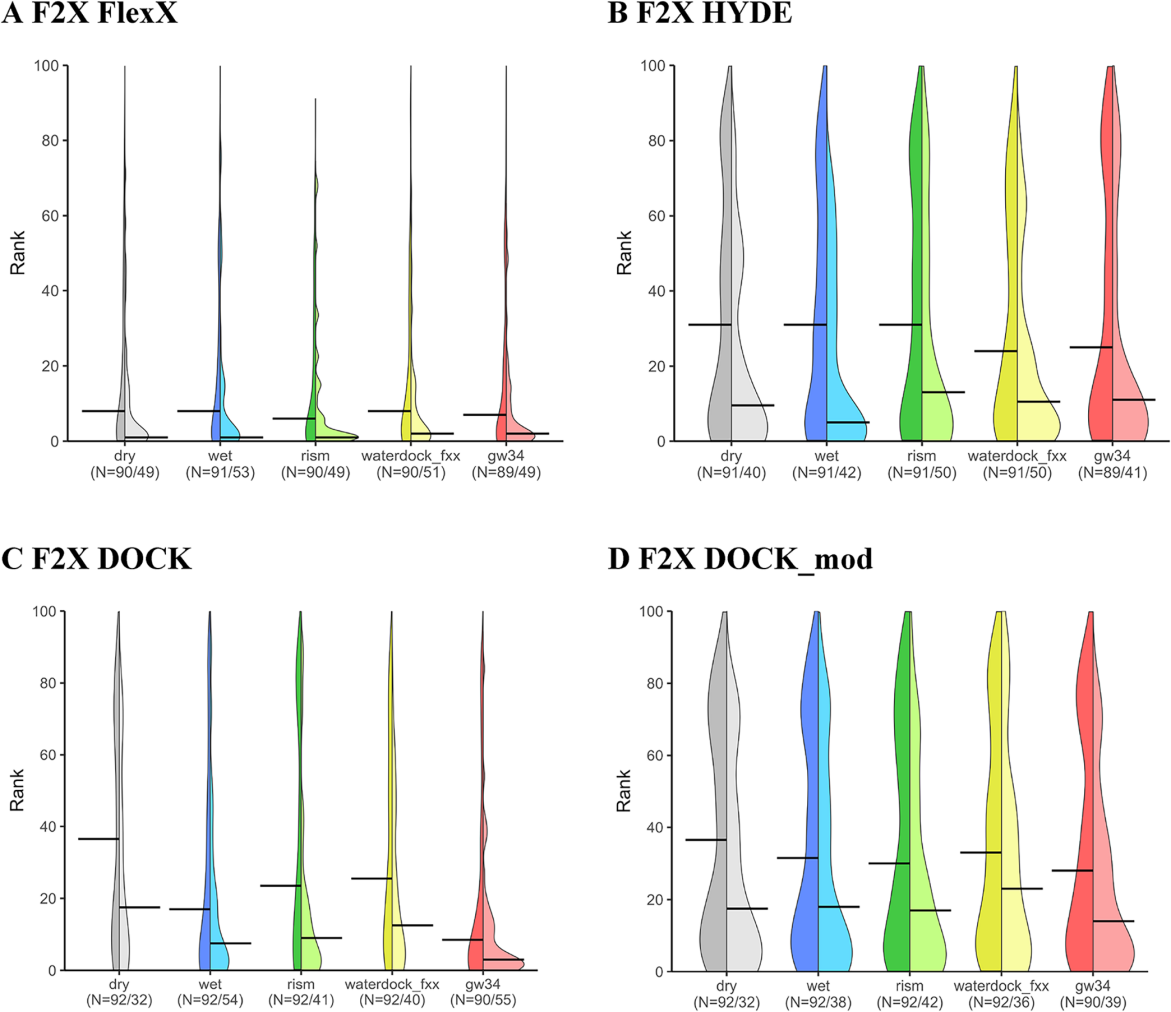
Violine plots of crystallographic fragment ligand ranks from LEADS-FRAG against F2X-decoys for water models *dry* (gray), *wet* (blue), *rism* (green), *waterdock_fxx* (yellow), and *gw34* (red) using (A) FlexX, (B) HYDE, (C) DOCK, and (D) DOCK_mod as docking programs. The left half of the violin includes all targets, the right half only those with correct redocking poses (RMSD ≤ 2.0 Å). The bold horizontal lines indicate the respective median.

**Table 2:** Third Quartiles of the Ranks of Binder vs Potential Nonbinder Discrimination with the F2X Decoys for the LEADS-FRAG Data Set (1 Ligand, 96 Decoys) (Number of Targets)^a^

		**rank of binders ≤ value in 75% of cases**				
		***dry***	***wet***	***rism***	***waterdock_fxx***	***gw34***
FlexX	all	31 (90)	30 (91)	30 (90)	27 (90)	26 (89)
	correct pose	12 (49)	15 (53)	7 (49)	14 (51)	10 (49)
HYDE	all	59 (91)	63 (91)	67 (91)	62 (91)	71 (89)
	correct pose	44 (40)	37 (42)	48 (50)	29 (50)	40 (41)
DOCK	all	70 (92)	45 (92)	72 (92)	60 (92)	36 (90)
	correct pose	46 (32)	24 (54)	21 (41)	39 (40)	13 (55)
DOCK_mod	all	70 (92)	70 (92)	67 (92)	65 (92)	68 (90)
	correct pose	46 (32)	51 (38)	46 (42)	46 (36)	36 (39)

a The ranks of the binders are ≤ the tabular value in 75% of the cases. The lower the value, the better. The first value includes all LEADS-FRAG entries, the value below only those with correctly predicted binding modes of the binders in the redockings (RMSDs ≤ 2.0 Å) = correct pose (number of data points).

For accurate ranking of molecules by the scoring function, successful posing is required to be “right for the right reason”.^119^ Therefore, ranking of the known ligand was compared with the F2X-decoys for cases where the predicted pose was correct (determined by redocking RMSD ≤ 2.0 Å). In all setups, the fragments from the crystal structures ranked higher when redocking poses were correct (Figure 2 and Table 2). For FlexX, correct poses allowed 75% of the ligands to be found in around the top 10% of the best-scoring molecules (Table 2). Still, the solvent models had minor effects on rank (Figure 2A). HYDE rescoring, DOCK, and DOCK_mod showed inferior results compared to FlexX, with 75% of the ligands found in the top 22–53% except for DOCK in combination with *gw34* in which 75% of cases the known ligands were found within the top 13% (Table 2). For DOCK using all predicted or experimental solvent sites, the ranking of the known ligand was improved most compared to *dry*. However, it is to be considered again that implementing all water molecules has also the highest bias toward the reference ligand (Figure 1 and S4). FlexX with different solvent models and the combinations of HYDE with *waterdock_fxx* as well as *gw34* with DOCK resulted to be most suitable in terms of redocking success rate and binder-decoy discrimination ability.

### Frag2Lead Data Set

While successful redocking is a prerequirement for every structure-based virtual screening, a more realistic scenario for FBDD would start from a target structure in complex with a different, most likely larger ligand. Likewise, once a fragment hit is identified, structure-based growing of this fragment is common during hit-to-lead optimization. To cover these real-world scenarios of FBDD, the study was expanded by cross-docking. Cross-docking of a fragment into a binding site of a target in complex with a different (bigger) ligand can reflect a fragment virtual screening campaign and serve as a surrogate task for synthon-based docking of ultralarge chemical spaces.^25,46–49^ Cross-docking of a fragment-derived lead molecule into a fragment binding site mimics the approach of fragment growing, hit-to-lead optimization, and the extension in synthon-based docking methods. We created a new benchmark data set based on published fragment-to-lead campaigns^86–91^ to evaluate these fragment-in-lead complex (FinL) or lead-in-fragment complex (LinF) approaches (Figure 3).

**Figure 3:**
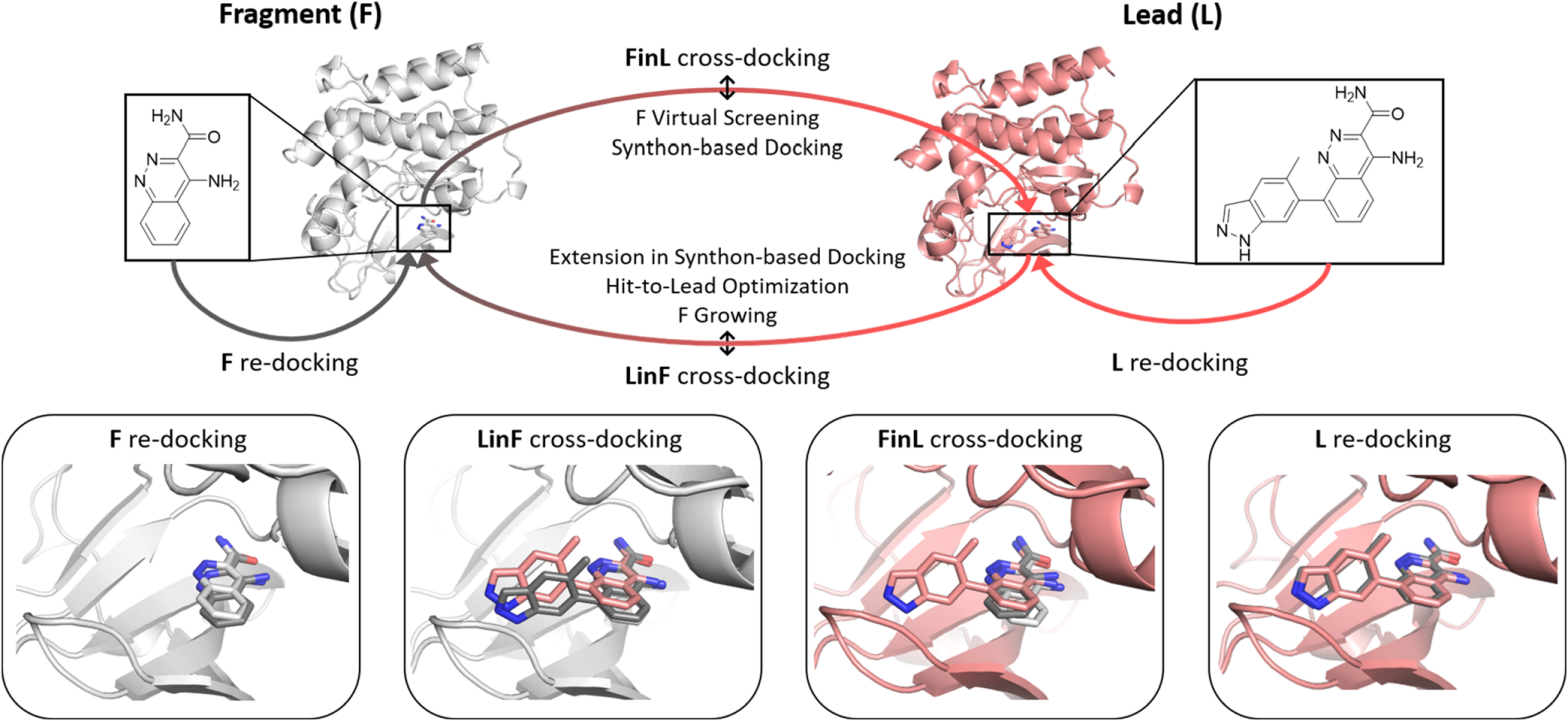
Schematic procedure of re- and cross-dockings shown with Bruton’s tyrosine kinase (entry 2015–01, fragment complex PDB 4ZLY,^120^ lead complex PDB 4Z3V^120^) of the Frag2Lead data set as an example. Protein-fragment complex crystal structures are illustrated in light gray, protein-lead complex crystal structures in salmon. The docking poses resulted from FlexX-*dry* Fragment (F) redocking, lead-in-fragment (LinF) template cross-docking, Fragment-in-Lead (FinL) cross-docking, and lead (L) redocking shown in gray. FinL and LinF cross-dockings are surrogates for real-world scenarios of FBDD and synthon-based VS. In the case of FinL cross-docking real-world scenarios, the screened fragments are usually structurally more distinct from the crystallographic reference ligand.

### Frag2Lead Data Set Redocking

The data set (in the following referred to as “Frag2Lead”) contains 103 fragment-protein and the corresponding lead-protein complexes from the PDB including four examples where one lead originates from two fragments by merging or linking, and one fragment grown into two different leads (Table S10). In contrast to the LEADS-FRAG data set with high resolutions, the Frag2Lead data set contains 60.2% (out of 201 unique entries) high-quality crystal structures (resolution ≤ 2.0 Å) (Table S10, compiled in Supporting Information). The average physicochemical properties of the fragments from Frag2Lead were similar to the LEADS-FRAG and F2X-Entry data sets (Table S6). The docking programs FlexX with and without HYDE rescoring and DOCK as well as DOCK_mod were used in combination with the water models *dry*, *wet*, *rism*, *waterdock_fxx*, and *gw34* (Table S10).

### Fragment Redocking

Success rates were slightly inferior for FlexX-based dockings and slightly superior in DOCK-based dockings (Table 3 and Figure S3) compared to the LEADS-FRAG data set (Table 1 and Figure S2). Fragment redocking performances with FlexX showed differences across the solvent models with *rism* (54%) being the most successful one compared to *dry* (43%) (Table 3 and Figure S3A) in contrast to the LEADS-FRAG data set (all success rates above 53%, Table 1). HYDE rescoring (Table 3 and Figure S3B) decreased the docking performances of FlexX fragment redockings (Table 3), but in less extent than for the LEADS-FRAG data set (Table 1). The best water model for HYDE fragment redocking was in both data sets *waterdock_fxx* with 52% (Table 3) and 54% for Frag2Lead and LEADS-FRAG (Table 1), respectively. For DOCK, all water models performed better than *dry* (44%) with *gw34* (66%) being the best one for fragment redocking followed by *rism* (59%) (Table 3 and Figure S3C) similar to the LEADS-FRAG data set (*dry* 35%, *gw34* 60%, Table 1). The fragment redocking performance of DOCK was superior to FlexX and HYDE dockings in general. The application of HYDE for solvation-site selection within DOCK_mod reduced the fragment redocking performances compared to DOCK (Table 3 and Figure S3D). The best water model for DOCK_mod was still *gw34* with a 51% success rate (Table 3). In total, the water models showed slightly improved fragment redocking performances compared to *dry* within the Frag2Lead data set in agreement with the results of the LEADS-FRAG data set.

**Table 3:** Fragment (Frag) and Lead Redocking Performances as Success Rate in % (Defined as RMSD ≤ 2.0 Å; Number of Data Points in Parentheses) of the Frag2Lead Data Set as Pose Prediction Success Rate (Based on All 103 Entries)^a^

	***dry***		***wet***		***rism***		***waterdock_fxx***		***gw34***	
	**Frag**	**Lead**	**Frag**	**Lead**	**Frag**	**Lead**	**Frag**	**Lead**	**Frag**	**Lead**
FlexX	43% (103)	58% (98)	51% (103)	57% (102)	54% (103)	58% (102)	50% (103)	57% (100)	50% (103)	58% (103)
HYDE	42% (103)	53% (99)	47% (103)	60% (102)	49% (103)	60% (102)	52% (103)	63% (100)	43% (103)	61% (103)
DOCK	44% (102)	45% (102)	52% (101)	57% (99)	59% (102)	56% (102)	55% (102)	58% (102)	66% (102)	72% (99)
DOCK_mod	44% (102)	45% (102)	49% (102)	53% (102)	47% (102)	59% (101)	46% (102)	58% (102)	51% (102)	50% (102)

a Visualization as violine plots is shown in Figure S3.

### Lead Redocking

Success rates (Table 3 and Figure S3E–H) were overall higher in comparison with the fragment redockings (Table 3 and Figure S3A–D) within the Frag2Lead data set. FlexX lead redockings indicated nearly no effect of the solvent models, with around 58% redocking success rates (Table 3 and Figure S3E) similar to the observations from the LEADS-FRAG data set (Table 1 and Figure S2A). Differently, for HYDE, DOCK, and DOCK_mod, improvements in lead redocking success rates arising from additional water molecules within the binding sites were found. Notably, DOCK includes more restrained water molecules than does FlexX during the docking process. For HYDE, the highest success rate was observed with *waterdock_fxx* (63% compared to *dry* 53%) (Table 3 and Figure S3F). Lead redockings shared the same best solvent model with the corresponding fragment redockings in 3 out of 4 cases and showed similar enhancing effects by the solvent models (Table 3). Lead redockings with DOCK had the best results with a 72% success rate with *gw34* (Table 3 and Figure S3G). All water models with DOCK showed improvements compared to *dry*, especially *rism* and *gw34*, which have the highest number of water molecules. The medians of the RMSD distributions were in all lead redocking cases (Figure S3E–H) under 2.0 Å except for DOCK *dry* (Figure S3G) and the identical DOCK_mod *dry* (Figure S3H). Compared to the fragment redocking, the success rate in pose prediction is higher for lead-like molecules as described previously.^17^

### Frag2Lead Data Set Cross-Docking

Using the Frag2Lead data set of target-fragment and corresponding target-lead pairs, cross-dockings were likewise performed using FlexX with and without HYDE rescoring and DOCK as well as DOCK_mod in combination with the water models *dry*, *wet*, *rism*, *waterdock_fxx*, and *gw34* (Table S10).

### Lead in Protein-Fragment Complex (LinF) Cross-Docking

LinF cross-docking success rates (Table 4 and Figure S5) illustrated a deterioration in pose prediction compared to fragment and lead redockings (Table 3 and Figure S3). LinF cross-dockings showed successful pose prediction in only around 20% of the cases and nearly no improvements by solvent models (Table 4 and Figure S5). *Dry* was in most cases the best docking setup. Especially, DOCK (without preselection of water molecules) failed to dock the bigger lead compounds into the water-filled protein-fragment complex binding sites due to the high space constraints introduced (Figures 1 and S4) and was excluded from further evaluations. For DOCK_mod, the implementation of crystallographic or predicted solvent models did not negatively affect the success rates of pose prediction as it does in the DOCK setup. LinF cross-docking results showed decreased docking performances compared to redockings. Additionally, LinF docking performances were not largely influenced by the different solvent models.

**Table 4:** Lead-in-Fragment Complex (LinF) Cross-Docking Performances as Success Rate in % (Defined as RMSD ≤ 2.0 Å; Number of Data Points in Parentheses) of the Frag2Lead Data Set Differentiating between Docking Performances Including All Data Points and Validated (val.) Ones, Only Including Data Points for Which the Corresponding Redocking (for LinF, the Fragment Redocking) Was Successful (RMSD ≤ 2.0 Å)^a^

**lead-in-fragment**		***dry***	***wet***	***rism***	***waterdock_fxx***	***gw34***
FlexX	all	22% (83)	18% (95)	21% (91)	17% (81)	19% (95)
	val.	25% (44)	21% (53)	25% (56)	25% (51)	25% (51)
HYDE	all	24% (83)	20% (95)	18% (91)	17% (82)	21% (96)
	val.	30% (43)	23% (48)	24% (50)	22% (55)	25% (44)
DOCK	all	18% (101)	12% (98)	0% (98)	7% (100)	4% (92)
	val.	20% (45)	15% (54)	0% (61)	5% (57)	1% (68)
DOCK_mod	all	18% (101)	10% (100)	12% (100)	11% (101)	15% (101)
	val.	20% (45)	12% (50)	13% (48)	13% (47)	15% (53)

a Visualization as violine plots is shown in Figure S5.

Under the assumption that a good pose prediction in redocking might correlate with better pose prediction in cross-docking of structurally similar ligands, LinF cross-dockings were re-evaluated for structures in which the corresponding redockings were successful. The comparison of LinF cross-docking performances including all data points with the ones having a previous successful fragment redocking showed slight improvements (Tables 4 and S10, Figure S5). For FlexX, the best solvent model changed from *dry*/*rism* to *waterdock_fxx* with a 26% success rate (increase of 9% through posing validation by redocking), with all solvent models performing similarly (Table 4). HYDE showed a similar increase of 7% in docking performance for the best model *dry* with 30% correctly predicted cross-docking poses (Table 4). DOCK_mod indicated a smaller improvement in LinF cross-docking performances (Table 4).

Even if this approach improved the results slightly, success rates of around 20–30% are insufficient to justify application in prospective fragment growing by docking or extension of a placed synthon in ultralarge library virtual screenings. However, often structural information on the fragment-bound target or a binding mode hypothesis is available as a starting point for fragment growing. This information can be used to guide docking using the fragment’s binding mode as a template or to define pharmacophore constraints as described previously.^3,25,46^ Implementation of such constraints for FlexX and HYDE cross-dockings with different solvent models was further explored (Figures 4 and S6, Table S11). Template docking improved the docking performances in all LinF cross-dockings (Figure 4) and approximately doubled the success rates in most of the cases (Table 5). FlexX LinF template cross-dockings reached a success rate of 52% while the best HYDE template cross-dockings showed around 43% success rate (Table 5). For solvent models under elucidation, still no general beneficial effect on success rates was observed, leaving *dry* as the best model (Table 5). Observed median RMSD values were below or close to 2.0 Å for FlexX LinF template cross-docking and for HYDE around 2.5 Å (Figure 4). Template docking approaches in LinF cases were found to be a beneficial strategy, which is more generally usable compared to the application of different solvent models. Using less constraints compared to template docking by applying one single pharmacophore feature per fragment as described previously,^3,25,46^ was also evaluated. The results using pharmacophore constraints for FlexX and HYDE LinF cross-dockings (*dry*), were inferior compared to template docking, but still showed some slight improvements for posing compared to unconstrained docking (Figure S6 and Table S11). Generating a specific pharmacophore model for a specific target informed by known structure–activity relationships (SAR) can improve docking performance while being less restrictive than template docking. In the framework of this study, however, LinF cross-dockings were most successful with a template-based approach without explicit solvent.

**Figure 4:**
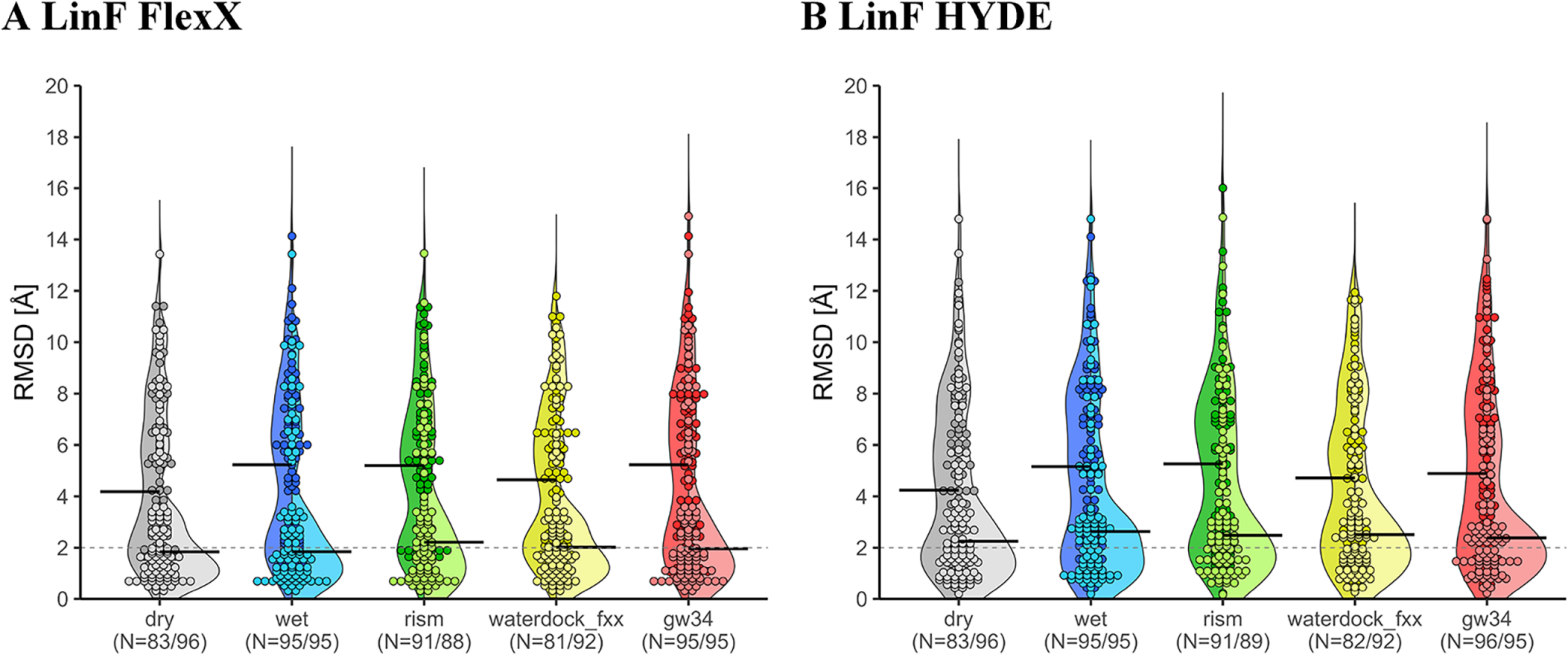
Violine plots of RMSD distributions for water model *dry* (gray), *wet* (blue), *rism* (green), *waterdock_fxx* (yellow), and *gw34* (red) using (A) FlexX and (B) HYDE for LinF cross-docking of the Frag2Lead data set. The left side of each violine plot implies cross-dockings; the right side represents cross-dockings with the respective fragment template. The number of data points is written under the solvent model label differentiating between the left and right sides. The bold horizontal bars indicate the median. The dashed line represents the 2 Å cutoff for correct posing.

**Table 5:** Comparison of Lead-in-Fragment (LinF) Cross-Docking Performances as Success Rate in % (Defined as RMSD ≤ 2.0 Å; Number of Data Points in Parentheses) of the Frag2Lead Data Set Using No Constraints, Pharmacophore (ph4, Only for the *dry* Receptor Setup) Constraints, and Template Docking

	***dry***			***wet***		***rism***		***waterdock_fxx***		***gw34***	
		ph4	temp.		temp.		temp.		temp.		temp.
FlexX	22% (83)	24% (75)	52% (96)	18% (95)	50% (95)	21% (91)	40% (88)	17% (81)	45% (92)	19% (95)	49% (95)
HYDE	24% (83)	26% (75)	43% (96)	20% (95)	36% (95)	18% (91)	34% (89)	17% (82)	35% (92)	21% (96)	37% (95)

### Fragment in Protein-Lead Complex (FinL) Cross-Docking

Similar to LinF cross-dockings (Table 4 and Figure S5), FinL cross-docking success rates (Tables 6 and S10) illustrated a deterioration in pose prediction accuracy compared to fragment and lead redockings (Table 3 and Figure S3). FinL cross-docking with FlexX in combination with *gw34* had the best results with a 40% success rate, but showed only minor improvements over *dry* with 35% (Table 6 and Figure S7A). In contrast, the HYDE FinL *dry* cross-docking success rate of 23% differed more from the best water model *rism* with 34% (Table 6 and Figure S7B). DOCK FinL cross-docking with *gw34* had a success rate for pose prediction of 39% while it was only 28% for *dry* (Table 6 and Figure S7C). The best solvent model for DOCK_mod was *waterdock_fxx* with a success rate of 34% (*dry* = 28%) (Table 6 and Figure S7D). Generally, lead redockings had at least 10% higher success rates across all solvent models (Table 3) compared to the corresponding FinL cross-dockings (Table 6). All medians of RMSD distributions of FinL cross-dockings were >2.0 Å (Figure S7A–D). Docking a fragment into a “bigger” lead binding site including water molecules created in the presence of the lead molecule again seemed to be of minor effect. The differences between the best solvent model and *dry* were similar or slightly smaller compared to lead redockings. Thus, the application of solvent models for FinL improves docking performance to a similar extent as in redockings, but the particular best solvent model may differ (Tables 3 and 6).

**Table 6:** Fragment-in-Lead (FinL) Cross-Docking Performances as Success Rate in % (Defined as RMSD ≤ 2.0 Å; Number of Data Points in Parentheses) of the Frag2Lead Data Set Differentiating between Docking Performances Including All Data Points and Validated (val.) Ones, Only Including Data Points for Which the Corresponding Redocking (for FinL Lead-re-docking) Was Successful (RMSD ≤ 2.0 Å)^a^

**Fragment-in-Lead**		***dry***	***wet***	***rism***	***waterdock_fxx***	***gw34***
FlexX	all	35% (103)	37% (103)	35% (102)	39% (103)	40% (103)
	val.	40% (60)	46% (59)	43% (60)	47% (59)	50% (60)
HYDE	all	23% (103)	19% (103)	34% (102)	24% (103)	29% (103)
	val.	35% (55)	23% (62)	32% (62)	29% (65)	35% (63)
DOCK	all	28% (102)	33% (101)	29% (102)	32% (102)	39% (102)
	val.	33% (46)	39% (59)	41% (58)	33% (60)	42% (74)
DOCK_mod	all	28% (102)	25% (102)	32% (101)	34% (102)	26% (102)
	val.	33% (46)	31% (55)	34% (61)	37% (60)	31% (52)

a Visualization as violine plots is shown in Figure S7.

Different from the LinF docking as a surrogate for a fragment growing approach, the FinL cross-docking cannot be solved by a template docking strategy (even though it would be technically possible in this specific data set). Usually, novel chemotypes distinct from the crystallographic reference ligand are to be discovered in fragment docking screens. As described for the LinF cross-docking (Table 4 and Figure S5), it was examined if the FinL cross-docking performance correlates with the quality of the lead redocking. The comparison of FinL cross-docking performances including all data points with only the ones having a previous successful lead redocking showed slight improvements (Table 6 and Figure S7). For the redocked validated entries using FlexX, the best solvent model remained *gw34* with 50% success rate (increase by 10%). The other solvent models *wet*, *rism*, and *waterdock_fxx* also performed better than *dry* (40%) with 46, 43, and 47% pose prediction success rate, respectively. All predicted solvent models showed a slightly higher benefit from validation of redockings compared to *dry* (Table 6 and Figure S7A). For HYDE, the differences between the solvent models and *dry* are smaller when only structures with preceding successful redocking were considered. *Dry* improved the most (difference 12%), reaching a 35% success rate and belonged to the best-performing solvent setups for HYDE together with *rism* (32%) and *gw34* (35%) (Table 6 and Figure S7B). The improvements for DOCK were similar across the setups except for *rism*, which achieved a 12% increase in docking performance reaching 41% (Table 6 and Figure S7C). *Gw34* remained one of the best-performing solvent models with a 42% success rate with DOCK (vs. 33% *dry*, Table 6). In the case of DOCK_mod, the improvements of limiting the data set of successfully redocked lead structures were similar, and the best setup remained *waterdock_fxx* with 37% (vs. *dry* 33%, Table 6 and Figure S7D). The best-performing combination of all setups was FlexX with *gw34* (and *waterdock_fxx*), showing successful pose prediction for FinL cross-docking of 40% before and 50% after limiting to successful lead redocking cases (39% and 47% for *waterdock_fxx* without and with validation by redocking, respectively).

Cross-docking success rates were still lower than fragment/lead redocking performances but showed beneficial effects when including solvent models. In general, the probability of a successful FinL cross-docking slightly increases if the lead redocking was already successful (Table 6). *Vice versa*, an unsuccessful redocking is a stronger predictor for an unsuccessful cross-docking (Figure S7E and Table S12, true negative rate 0.6–0.9). Different from the LEADS-FRAG data set, the compiled target-fragment and corresponding target-lead pairs are not exclusively high-resolution structures (average resolution of 2.0 Å, Table S10). The dependency of the crystal structure’s resolution on the docking performance of the solvent model *wet* was elucidated for re- and cross-dockings, but no correlation was found (Figure S8).

### Binder-Decoy Discrimination

Like for the LEADS-FRAG data set (Figure 2 and Table 2), we used the F2X-Entry fragments as decoys to analyze the ability of the solvent models to improve discrimination of binder from potential nonbinder fragments in molecular docking. Fragment redocking (Tables 7 and S13, Figure S9A) and FinL cross-docking (Tables 7 and S13, Figure S9B,C) with FlexX were tested. FlexX was the most accurate and consistent program in fragment redocking (Tables 1 and 3, Figures S2 and S3) as well as fragment ligand from F2X-decoy discrimination of the LEADS-FRAG data set (Figure 2 and Table 2) and FinL cross-docking (Tables 6 and S7). Rankings of the fragments of the Frag2Lead data set within the F2X-decoys by docking score were improved when only including entries with previously correctly predicted binding modes (RMSD ≤ 2.0 Å) (Table 7 and Figure S9). This effect was more pronounced for fragment redockings than for FinL cross-dockings (Table 7) indicative of a better-defined binding site and less structural changes. The rank distributions (Figure S9A,B) and the third rank-quartiles (Table 7) for fragment binders with correct poses indicated that 75% of the binders are ranked among the top 20 of best scored molecules for fragment redocking and top 30 for FinL cross-dockings. Like for the LEADS-FRAG data set, solvent models had no consistent effects on the ranking. Rank distributions of fragments against F2X decoys for the LEADS-FRAG data set (Table 2) were slightly better than for Frag2Lead (Table 7). In the case of FinL cross-docking, validation via FinL RMSDs might not be suitable for real scenarios since these poses are likely unknown. Therefore, the rank distributions for FinL cross-docking with a successful lead redocking were analyzed (Figure S9C and Table 7). The third quartiles of the ranks showed slight improvements for *dry* and *wet*, and moderate improvements for *rism*, *waterdock_fxx*, and *gw34* (Table 7), respectively.

**Table 7:** Third Quartiles of the Ranks (the Lower, the Better) of Binder vs Potential Nonbinder Discrimination with F2X Decoys for the Frag2Lead Data Set Out of 103 Possible Rank Positions (Number of Cases)^a^

		**rank of binders ≤ value in 75% of cases**				
**FlexX**	**selection**	***dry***	***wet***	***rism***	***waterdock_fxx***	***gw34***
Frag redocking	all	30 (103)	30 (103)	24 (103)	24 (103)	23 (103)
	correct pose	18 (44)	27 (53)	19 (56)	19 (51)	19 (51)
FinL cross-docking	all	35 (103)	35 (103)	40 (102)	43 (103)	39 (103)
	correct pose	29 (36)	32 (38)	35 (36)	26 (40)	30 (41)
	val.	32 (60)	33 (59)	31 (60)	29 (59)	31 (60)

a The ranks of the binders are ≤ the tabular value in 75% of the cases. First value includes all Frag2Lead target entries, the value below only those with right positioning of the binder of the particular docking (either FinL or fragment docking, RMSDs ≤ 2.0 Å) = “correct pose”. For FinL, validation based on right positioning of the lead binder in lead redocking (val.) was also considered. Visualization as violine plots is shown in Figure S9.

### Consensus Docking

There was no clear evidence for a particular solvent model to generally improve fragment docking performance in terms of pose prediction accuracy (Tables 1 and 3, Figures S2 and S3) or ranking binders against the F2X-decoys (Tables 2 and 7, Figures 2 and S9). However, we observed a huge variation between the solvent model impact on docking for specific targets (Tables S7 and S10). Thus, a consensus docking using multiple solvent models might have beneficial effects, similarly to the selection of multiple target structures in an ensemble docking,^121,122^ or multiple docking tools or scoring functions for consensus docking and scoring.^121,123–126^ In a “pick-best” approach, pose prediction of fragment and lead redocking as well as FinL and LinF cross-dockings were re-evaluated for the LEADS-FRAG and Frag2Leads data sets (Table 8, Figures S10 and S11). The success rates of fragment and lead redockings for both data sets (LEADS-FRAG and Frag2Lead) using the “pick-best” consensus over multiple solvent models exceeded the success rates of each single solvent model with 59–88% correctly predicted binding modes (Tables 1, 3, and 8). For all three redockings, DOCK, which maintains the highest number of water molecules, yielded the best results (with 79–88%), followed by HYDE, FlexX, and DOCK_mod (Table 8). Notably, HYDE surpassed FlexX, indicating a higher sensitivity to the selected solvent model. The consensus model over both, all solvent models and all docking programs, achieved redocking success rates above 90% and RMSD averages/median values below 1.0 Å (Table 8 and Figure S10A–C).

**Table 8:** Consensus Success Rate (RMSD ≤ 2.0 Å), Average and Median of the RMSD Values When Picking the Best RMSD Per Entry among the Different Water Models (*dry*, *wet*, *rism*, *waterdock_fxx*, *gw34*) for Both Data Sets (LEADS-FRAG and Frag2Lead) and the Different Redocking and Cross-Docking Categories (for LinF, FlexX, and HYDE, Template Docking Was Included)^a^

**data set**	**docking category**	**docking software**	**success rate** (%)	**average** [Å]	**median** [Å]
**LEADS-FRAG**	Fragment redocking	FlexX	68	1.6	1.0
		HYDE	71	1.4	0.7
		DOCK	79	1.2	0.4
		DOCK_mod	59	2.6	1.2
		**consensus**	**92**	**0.6**	**0.3**
**Frag2Lead**	Fragment redocking	FlexX	71	1.7	1.1
		HYDE	81	1.4	0.9
		DOCK	86	1.0	0.4
		DOCK_mod	66	2.3	0.8
		**consensus**	**97**	**0.5**	**0.3**
	Lead redocking	FlexX	77	1.8	1.0
		HYDE	82	1.5	0.7
		DOCK	88	1.1	0.4
		DOCK_mod	75	2.1	0.7
		**consensus**	**96**	**0.6**	**0.3**
	Fragment-in-Lead cross-docking	FlexX	50	3.0	2.1
		HYDE	50	2.7	2.1
		DOCK	60	2.3	1.4
		DOCK_mod	52	3.0	1.8
		**consensus**	**80**	**1.4**	**0.9**
	Lead-in-Fragment cross-docking	FlexX	33	3.9	2.9
		+template	60	2.5	1.6
		HYDE	37	3.9	2.9
		+template	63	2.4	1.5
		DOCK_mod	25	5.2	5.0
		**consensus**	**51**	**2.8**	**1.9**
		**+template**	**72**	**1.9**	**1.3**

a Consensus model over water models and docking software written in bold. Visualization as violine plots is shown in Figure S10.

For the more challenging cross-dockings, the solvent consensus model within and across the different docking setups also exceeded the previous docking performances (Tables 4 and 8). The “pick-best” FinL FlexX consensus solvent model achieved 50% success rate (Table 8), the same as FinL FlexX *gw34* and similar to *wet* and *waterdock_fxx* with preselected lead redockings (Table 4). This reveals that FlexX in combination with *gw34*, *wet*, or *waterdock_fxx* is a good option for FinL cross-dockings. DOCK revealed even higher success rates in a consensus solvent model with 60% for FinL cross-dockings, however, likely caused by the strong bias from a large number of solvent molecules included in the receptor presentation (Figures 1 and S4). The FinL consensus docking over different solvent models showed 50–60% success rates increasing to an 80% success rate for consensus over all docking tools and an average RMSD of 1.4 Å (Table 8).

In the case of LinF cross-dockings, success rates of FlexX (33%) and HYDE (37%) were slightly better than those of DOCK_mod (25%). When template dockings were included for FlexX and HYDE, it improved to 60 and 63% of correctly predicted poses, respectively. The overall consensus model reached a success rate of 51% without and 72% with template docking inclusion and an average RMSD of 2.8 and 1.9 Å for the LinF cross-docking, respectively (Table 8).

The detailed composition of the “pick-best” consensus model over water models and docking software (Table 8) was different for different docking categories (Figure S11A–F). Fragment and lead redocking of the Frag2Lead data set showed similar distributions with DOCK covering more than 50% occurrence, with *rism* and *gw34* taking the biggest part within DOCK probably caused by the strong bias formed by a high number of water molecules (Figures 1, S4, and S11B,C). The consensus model of fragment redocking of the LEADS-FRAG data set implied a higher proportion of HYDE (a quarter, Figure S11A) than in the Frag2Lead data set (Figure S11B), but with DOCK still forming the biggest part with almost 50%. *Rism*, *wet*, and *gw34* were mostly represented within DOCK (Figure S11A). For FinL cross-dockings, DOCK and HYDE showed similar shares with both accounting for one-third of the consensus model (Figure S11D). Here, the docking software as well as the solvent models are more equally distributed (Figure S11D) compared to the redockings of the Frag2Lead data set (Figure S11A–C). In LinF cross-dockings excluding template dockings, the shares of FlexX, HYDE, and DOCK_mod were similar (Figure S11E). When including template docking, LinF cross-dockings were dominated by FlexX and HYDE, occupying more than two-thirds of the pie together (Figure S11F). Notably, FlexX in combination with *dry* template docking had the biggest share (Figure S11F). While such “pick-best” consensus models are suitable to evaluate redockings, for prospective applications (Figure 3) in fragment growing (surrogate LinF) or identification of novel fragment binders (surrogate FinL), the correct answer for pose prediction might not be known in advance and the RMSD value cannot be determined. Selection of a solvent model (and docking software) successful in redocking, however, did not imply better predictions for cross-docking (Table S5 and Figure S12).

## Discussion

The effect of explicit crystallographic or predicted water molecules on the docking performance of fragments was investigated by redocking and cross-docking fragments into binding sites occupied by larger ligands (fragment-in-lead cross-docking, FinL) and *vice versa* (lead-in-fragment cross-docking, LinF). These cross-dockings reflect real-world scenarios (Figure 3) of protein-fragment docking virtual screenings (FinL) and the initial fragment placement in synthon-based dockings of ultralarge chemical spaces. Lead-in-fragment cross-dockings (LinF) of a fragment-derived lead molecule into a fragment binding site can mimic fragment growing, hit-to-lead optimization, and the extension in synthon-based docking methods. We used two different data sets to accomplish our studies. The previously reported LEADS-FRAG data set^24^ was used to evaluate fragment redockings. For the cross-docking studies, we created a new benchmark data set, labeled Frag2Lead, which was extracted from published fragment-to-lead campaigns.^86–91^ The Frag2Lead data set (Figure S13, Table S10, Supporting Information) contains 103 fragment-protein and corresponding aligned lead-protein complexes with an overall average resolution of 2.0 Å (out of 201 unique entries, four lead and one fragment entry appear twice). Our comprehensive statistical evaluation mainly focused on docking without water molecules (*dry*), crystallographic water molecules (*wet*), and predicted water molecules using 3D-*RISM* (*rism*), docking-placed water molecules using FlexX (*waterdock_fxx*), and the machine learning model Galaxywater-CNN (*gw*). Dockings were conducted using four docking tool variations. FlexX^80^ with and without HYDE^81,82^ rescoring and DOCK^16^ as well as DOCK with water molecules preselected by HYDE based on their interaction profiles (DOCK_mod).

Our studies did not identify a single superior combination of docking program and solvent model. Instead, we observed a high variability of solvent model’s impact on the targets across the data sets and across the different docking tools and tasks. However, overall water models performed slightly better in fragment redocking than without (*dry*) explicit solvent molecules within the binding sites (Tables 1 and 3). This effect was more pronounced in dockings considering a higher number of water molecules such as DOCK compared to DOCK_mod or HYDE and FlexX. However, such a strong bias by a high number of water molecules also led to a disadvantage in cross-dockings.

To specifically address this question, we compiled the Frag2Lead data set of corresponding protein-fragment and protein-lead complexes. Generally, cross-docking accuracy (Tables 4 and 6) was lower than for redocking fragments or lead-like molecules (Tables 1 and 3). Especially, when docking a larger ligand, like a lead derived from a fragment, into the binding site of a protein-fragment complex (LinF, Table 4), too strong solvent constraints prevent proper posing. Rather than relying on improved docking accuracy by the implementation of further solvent information, alternative strategies have turned out to be more reliable. Template-based docking (using the fragment as the template molecule) or the addition of fragment-derived pharmacophore constraints can improve docking accuracy for the respective lead-like structures (Tables 5, S11, and Figure S6).

The more challenging cross-docking task turned out to be fragment docking into a larger binding site (FinL, Table 6). Usually, this task cannot be simplified by template docking, as novel scaffolds are to be identified. Even though the dynamic effects of protein–ligand interactions were of minor relevance as the receptor for fragment docking was in complex with a lead-like structure derived from the respective fragment, correct poses could only be obtained in 19–40% of cases depending on the docking tool and water model (Table 6). This value increases to up to 50% for cross-dockings when limiting to cases with a successful preceding redocking of the lead ligand. However, this also indicates that, even though redocking is considered the minimal docking validation, it is not a strong predictor for a successful cross-docking (Table 6). However, an unsuccessful redocking well predicts the setup to be unsuitable for cross-docking (true negative rate 0.6–0.9, Table S12 and Figure S7E).

While there was no single docking and water model combination found to be superior over the others, there was a huge target-wise variation. Thus, we evaluated a “pick-best” consensus docking using multiple solvent models for all data sets. Success rates of fragment and lead redockings exceeded previous results using the “pick-best” consensus docking approach. Further, consensus over both, solvent models and docking software, achieved success rates of over 90% in fragment and lead redockings (Table 8). Cross-docking performances improved using the consensus model, reaching up to 80% for FinL and 72% for LinF (including template docking; Table 8). However, such a “pick-best” approach is not realistic for FinL cross-dockings since the correct pose might not be known *a priori*. Again, using redocking for the selection of the most suitable docking tool and water model combination for cross-docking led to only minor improvements (Table S5 and Figure S12). This is in agreement with the observation that redockings and the corresponding cross-dockings do not share the same best software and solvent model combination (Figure S11).

Besides pose prediction, molecular docking has to discriminate binders from nonbinders (or decoys) by its scoring function. Using the structurally diverse F2X-Entry set as decoys, binder fragments were usually (third quartile, 75% of cases) ranked within the first third (FlexX) or half (HYDE, DOCK, DOCK_mod) of the data set or better when using only correct binding modes to be “right for the right reason”^119^ (Tables 2 and 7). Again, a slight trend of improved ranking by water molecules by some models compared with *dry* receptors was found. For the FinL receptors, binder fragments were also ranked slightly higher, when the pose of the respective lead compound was correctly predicted in a preceding redocking.

## Limitations

Our comprehensive study on the effect of including water molecules during fragment docking on the docking performance faces some limitations and assumptions. The solvent selection and dockings were conducted via an automated workflow to minimize human bias introduced by user intervention. However, specific knowledge about the target’s binding site was not acquired or used. It is known that water networks in protein binding sites can vary between apo and holo structures and even depend on which type of ligand is bound.^53,54^ In a real-world drug discovery campaign, all information about the binding site, including key interactions, conserved hydration sites among different apo or complex structures, and structural variations, may be implemented. Especially, knowledge about target- and/or ligand-specific, key water molecules can be beneficial for docking.^71,112^ Further, the selection of water molecules for docking used the simplified approach of FlexX/HYDE. This approach relies on the number of interactions a water molecule can form with its surroundings rather than an energetic term. Indirectly, this was implemented by a cutoff of docking scores (*waterdock* and *waterdock_fxx*), the *gw*-score or the energy estimate of 3D-*RISM*. Different, likely stricter cut-offs may lead to different docking results. The FinL cross-docking approach assumed that a fragment is docked into a fragment-derived lead complex, which is an unlikely event in drug discovery. Usually, fragment screening aims to find novel scaffolds different from already known lead structures. Target dynamics and conformational changes, which might occur from apo or structurally distinct holo structures, were neglected by this FinL approach. By doing so, we focused only on the isolated challenge of solvent inclusion for fragment docking while ignoring the target dynamics.

## Conclusion

In summary, the results highlight once more the importance of proper model validation. The inclusion of water models during docking was found to be beneficial in fragment and lead redocking and FinL and LinF cross-docking. But the preferred combination of docking tool and water model differed across the different docking tasks and targets. Testing multiple setups or development of a consensus model over not only docking tools but also multiple solvent models is advised. A successful redocking as the minimal docking setup validation already increases the probability of a successful cross-docking and hence general pose prediction. For FinL cross-dockings, representing fragment virtual screenings and the initial fragment docking in synthon-based dockings, solvent models performed in most cases better than using *dry* receptors. In contrast, LinF cross-dockings, representing fragment growing or extensions in synthon-based dockings, benefit the most when using a template-based method or the inclusion of pharmacophore constraints rather than from different solvent models. However, such constraints can limit the structural diversity of potential hits. This is no limitation for fragment growing but might be for scaffold hopping approaches.

Further, a consensus docking using multiple solvent models, which were selected by the corresponding redocking, was suitable for LinF cross-dockings, too. We emphasize that inclusion of a high number of water molecules during docking puts too many constraints on the binding sites and should be considered with care. Including all available water molecules might increase redocking success rates but is not rational for prospective screenings. In contrast, only including target-specific key water molecules might be a reasonable alternative and showed promising results in multiple cases.^71,112^ However, more information about conserved and important water sites is required than was implemented in this automated workflow. Dealing with hydration within binding sites is only one challenge in fragment docking. The accuracy of scoring fragments is still a main challenge due to the small size and low binding free energies of the fragment binding to its target.^12,13^ While besides target dynamics, “*water is to blame for* [nearly] *everything*”^127^ that can be challenging in molecular docking, it seems insufficient to “*just add water*”^52^ for fragment docking. Rather “*combining things of both worlds*”^123^ like using consensus over multiple solvent models and docking tools seems to be a more promising approach. Lastly, the presented Frag2Lead data set is made available for further retrospective cross-docking studies and model development as an addition to already available data sets like LEADS-FRAG^24^ for fragment redocking. It allows one to mimic real-world scenarios of FBDD, fragment docking, and synthon-based VS.

## Supplementary Material









## Data Availability

Molecular dockings were performed with FlexX, HYDE, and DOCK using the cited versions in the Materials and Methods section. Additionally, QUACPAC molcharge and OMEGA from OpenEye Applications, MOE, and MGLTools within AutoDockTools using the cited versions in the Materials and Methods section were used for the described steps in receptor and ligand preparation. Pharmacophore constraints were created within SeeSAR with the cited version. AmberTools22 and NAMD were used for MD simulation preparation and execution. For binder-decoy discrimination, the freely available F2X-Entry data set was used. LEADS-FRAG data set is freely available. Frag2Lead data set with protein-fragment and corresponding protein-lead complexes as well as docking raw data are provided in the Supporting Information. The 3D-*RISM* method was used within MOE. Galaxywater-CNN (https://github.com/seoklab/GalaxyWater-CNN, Web server: https://galaxy.seoklab.org/cgi-bin/submit.cgi?type=GWCNN_INTRO) and Waterdock2.0 (https://github.com/bigginlab/WaterDock-2.0) are freely available. The *waterdock_fxx* python scripts (flexx-waterdock.py and topwater.py) and usage documentation are available in the Supporting Information (Extended Material and Methods).

## Notes

Detailed results of re- and cross-dockings, discrimination of fragment binders from F2X decoys, Frag2Lead data set information, and *waterdock_fxx* implementation (Tables S1–S6) (Figures S1–S14). Additional References (PDF)
LEADS-FRAG, Frag2Lead docking raw data and data set information analyses (Tables S7–S13) (XLSX)
Frag2Lead data set with protein-fragment and corresponding protein-lead complexes (ZIP containing PDB and SDF) (ZIP)
*waterdock_fxx scripts* (ZIP containing PY) (ZIP)

## Abbreviations

3D-*RISM*: three-dimensional reference interaction site model
AUC: area under curve
BCL: b-cell lymphoma
CNN: convolutional neural network
FBDD: fragment-based drug discovery
FBS: fragment-based screening
FDA: U.S. Food and Drug Administration
FinL: fragment-in-lead
frag: fragment
HTS: high-throughput screening
LE: ligand efficiency
LinF: lead-in-fragment
MD: molecular dynamic
PDB: protein databank
RMSD: root-mean-square deviation
ROC: receiver-operating characteristic
SAR: structure–activity relationship
SBDD: structure-based drug design
val.: validated
VS: virtual screening
