# Supplementary material for: Solvent-Site Prediction for Fragment Docking and Its Implication on Fragment-Based Drug Discovery
Source: J Chem Inf Model. 2025 Nov 24;65(23):12959–77. doi: 10.1021/acs.jcim.5c02352 (PMC12690578; doi:10.1021/acs.jcim.5c02352)
Supplement: Supplementary file 1 [file ci5c02352_si_001.pdf]

Supporting Information:

Solvent-Site Prediction for Fragment Docking and its Implication on Fragment-Based Drug Discovery

*Laura Almena Rodriguez[1], Vera A. Spanke[1,2], Christian Kersten[1, 3] **

[1] Institute of Pharmaceutical and Biomedical Sciences, Johannes Gutenberg-University Mainz, Staudingerweg 5, 55128 Mainz, Germany.

[2] Department of General, Inorganic and Theoretical Chemistry, and Center for Molecular Biosciences Innsbruck, University of Innsbruck, 6020 Innsbruck, Austria.

[3] Institute for Quantitative and Computational Biosciences, Johannes Gutenberg-University, BioZentrum I, Hanns-Dieter-Hüsch Weg 15, 55128 Mainz, Germany.

*Corresponding author: kerstec@uni-mainz.de

Content

1. Docking Analysis: Figures S1 – S12, Tables S1 – S5 2
2. Frag2Lead Dataset: Figure S13, Table S6 16
3. Extended Material and Methods – Implementation of waterdock_fxx: Figure S14 35
4. References 37

1. Docking Analysis: Figures S1 – S12, Tables S1 – S5

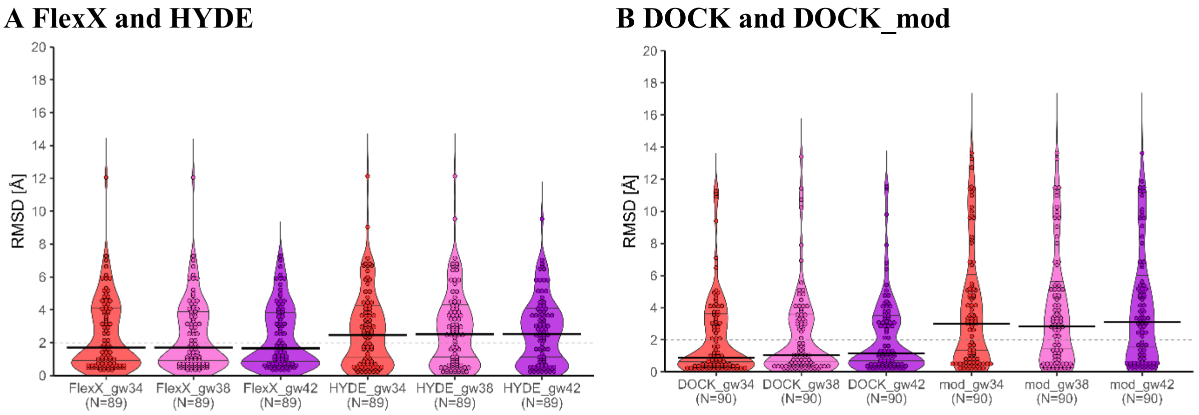

Figure S1: Comparison of different Galaxywater-CNN (*gw*) score cut-offs. Violine plots of RMSD distributions for water model *gw* using **A**) FlexX and HYDE, **B**) DOCK and DOCK_mod (mod) for re-dockings of the LEADS-FRAG dataset with *gw* score cut-off ≥34 (red), ≥38 (pink) and ≥42 (purple). The bold horizontal bars indicate the median.

Table S1: LEADS-FRAG dataset re-docking success rates in % (number of datapoints) defined as predicted binding modes with RMSD ≤ 2.0 Å compared to crystal structure using hydrated receptors predicted by Galaxywater-CNN (*gw*) with different cut-off values.

	Water model		
**Software**	**gw34**	**gw38**	**gw42**
**FlexX**	53% (89)	53% (89)	54% (89)
**HYDE**	45% (89)	42% (89)	40% (89)
**DOCK**	60% (90)	61% (90)	59% (90)
**DOCK_mod**	42% (90)	40% (90)	38% (90)

Table S2: LEADS-FRAG dataset re-docking success rates in % (number of datapoints) defined as predicted binding modes with RMSD ≤ 2.0 Å compared to crystal structure using *waterdock* and *waterdock_fxx* as water placing method.

	Water model	
**Software**	**waterdock**	**waterdock_fxx**
**FlexX**	52% (91)	57% (91)
**HYDE**	43% (91)	54% (91)

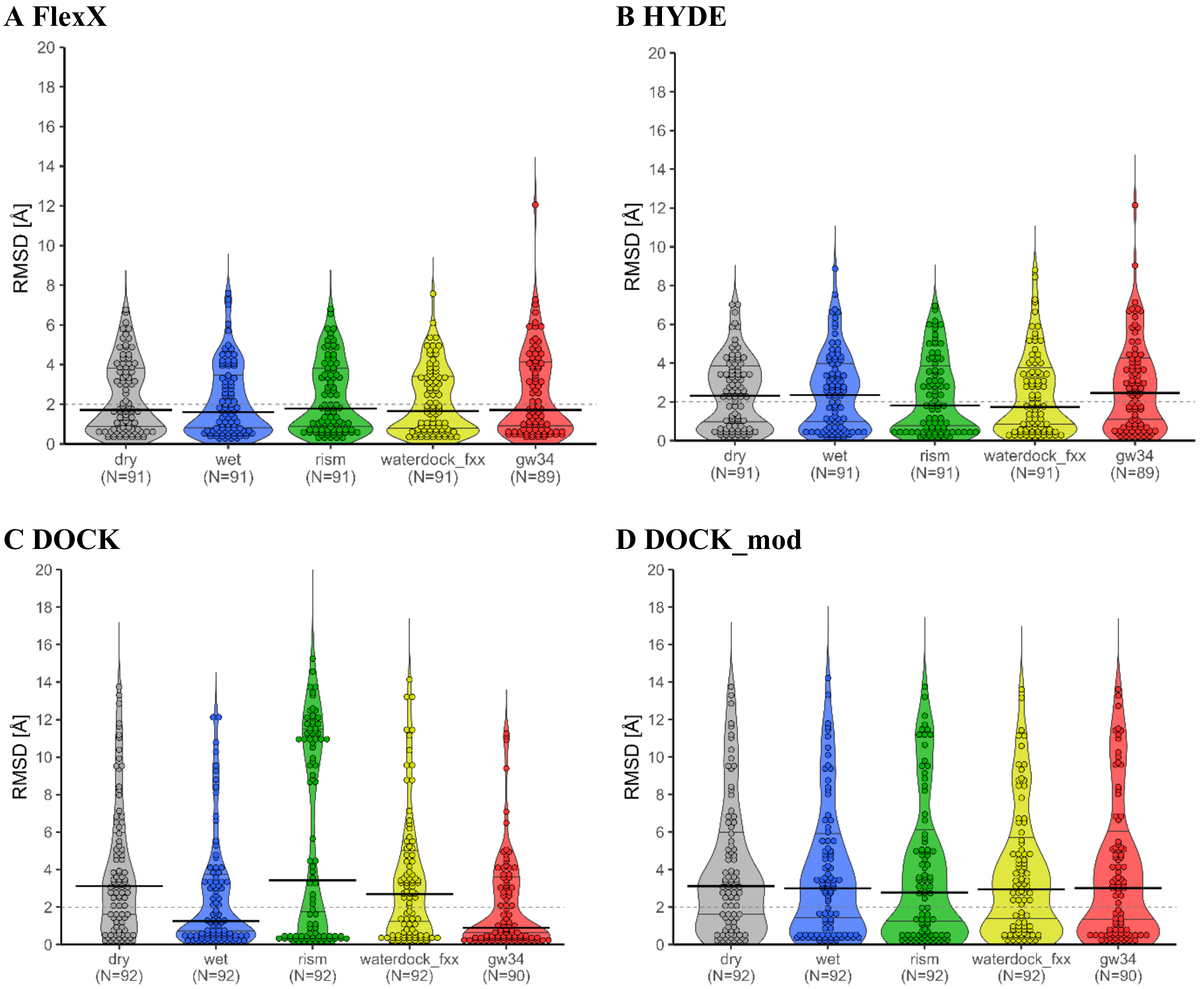

Figure S2: Violine plots for re-docking RMSD values for different solvent models: *dry* (grey), *wet* (blue), *rism* (green), *waterdock_fxx* (yellow) and *gw34* (red) using **A)** FlexX, **B)** HYDE, **C)** DOCK and **D)** DOCK_mod as docking programs for the LEADS-FRAG dataset. The bold horizontal lines indicate the median values and the thin horizontal lines within the violine body represent the first and third quartile. The dotted line at 2.0 Å indicates the separation criterium for a successful re-docking.

Table S3: LEADS-FRAG dataset re-docking RMSD values in Å including SPAM method for FlexX. The PDB entries were chosen based on five categories. 1: Docking performance of crystallographic water *wet* and predicted water sites with *rism* outperforms *dry* docking. 2: Docking performance of crystallographic water *wet* outperforms predicted waters with *rism* and *dry* docking. 3: Docking performance of predicted waters with *rism* outperforms crystallographic water *wet* and *dry* docking. 4: Docking performance of crystallographic water *wet* and *dry* docking outperforms predicted waters with *rism*, so the prediction fails. 5: Docking performances of all three methods were unsuccessful (RMSD ≤2.0 Å). Two entries per category were selected.

Category	PDB Entry	wet	dry	rism	SPAM
**1**	2DZA	2.6	5.5	1.1	5.5
	4LVB	1.1	3.2	1.1	3.2
**2**	3MZ9	0.4	1.6	3.9	4.4
	4K5Z	1.5	3.0	4.9	4.9
**3**	4RLQ	2.9	3.6	1.0	3.6
	4JYM	4.0	5.8	2.5	5.8
**4**	3SRB	0.8	0.8	5.3	0.8
	4KLW	0.4	0.4	3.4	0.4
**5**	1Y2C	3.9	3.4	4.1	3.7
	3CHC	7.6	6.6	6.6	6.6

Table S4: LEADS-FRAG dataset re-docking RMSD values in Å including SPAM method for DOCK The PDB entries were chosen based on five categories. 1: Docking performance of crystallographic water *wet* and predicted water sites with *rism* outperforms *dry* docking. 2: Docking performance of crystallographic water *wet* outperforms predicted waters with *rism* and *dry* docking. 3: Docking performance of predicted waters with *rism* outperforms crystallographic water *wet* and *dry* docking. 4: Docking performance of crystallographic water *wet* and *dry* docking outperforms predicted waters with *rism*, so the prediction fails. 5: Docking performances of all three methods were unsuccessful (RMSD ≤2.0 Å). Two entries per category were selected.

Class	PDB Entry	wet	dry	rism	SPAM
**1**	1Y2C	0.2	6.9	0.3	4.7
	5D7X	1.8	4.9	1.9	5.0
**2**	1Q11	0.6	5.3	10.8	2.3
	4K5Z	0.3	4.6	4.5	4.6
**3**	2DZA	3.8	2.6	0.2	1.3
	4JYM	2.4	2.4	0.5	2.5
**4**	3BU1	1.3	1.2	3.9	1.9
	3SRB	0.3	0.4	15.3	0.4
**5**	4OWU	9.3	9.4	11.0	7.0
	4RLQ	12.1	3.1	11.8	3.1

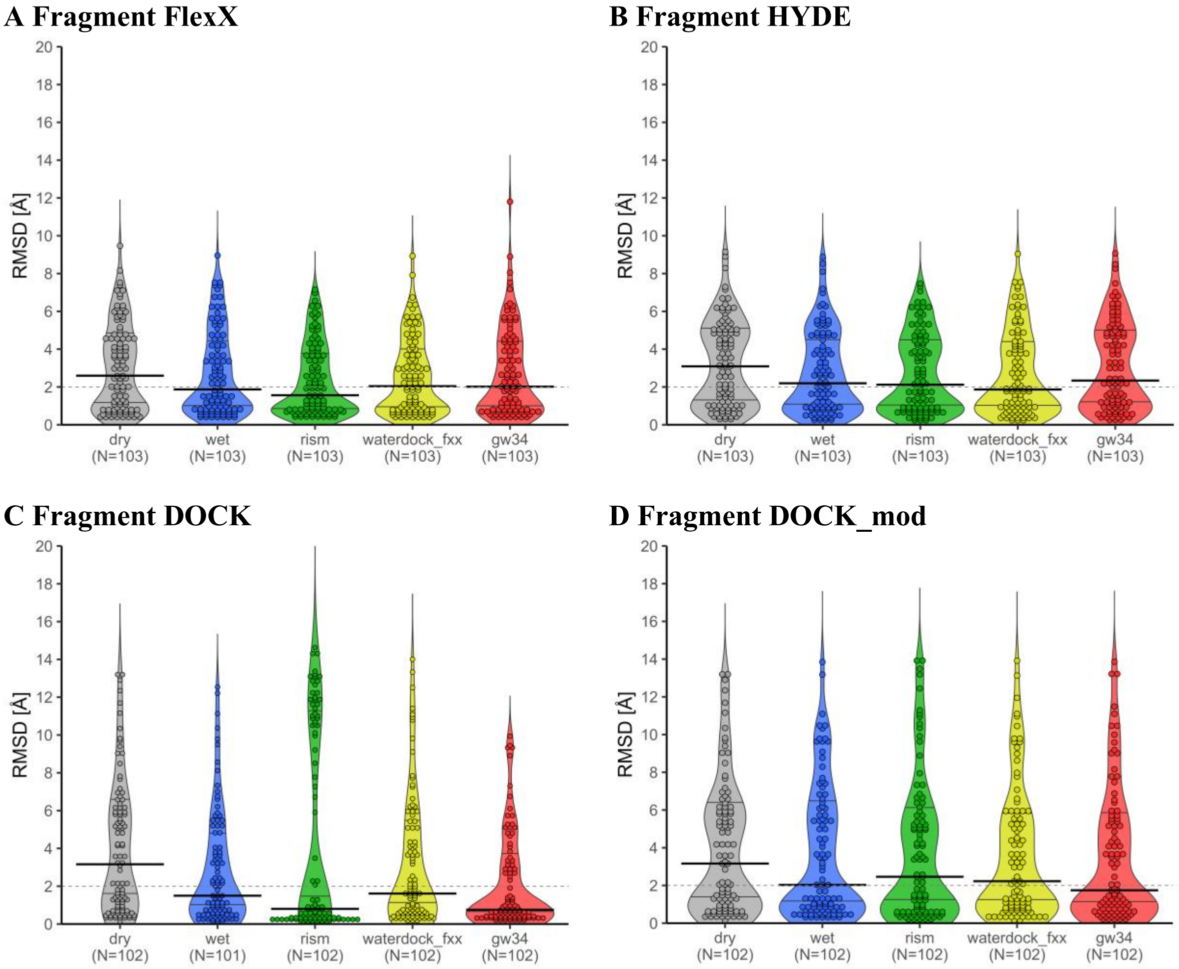

Figure S3: Violine plots of RMSD distributions for water model dry (grey), wet (blue), rism (green), waterdock_fxx (yellow) and gw34 (red) using **A**) FlexX, **B**) HYDE, **C**) DOCK and **D**) DOCK_mod as docking programs for fragment re-docking of the Frag2Lead dataset and **E**) FlexX, **F**) HYDE, **G**) DOCK and **H**) DOCK_mod as docking programs for lead re-docking of the Frag2Lead dataset. The bold horizontal bars indicate the median and the thin horizontal lines within the violine body represent the first and third quartile.

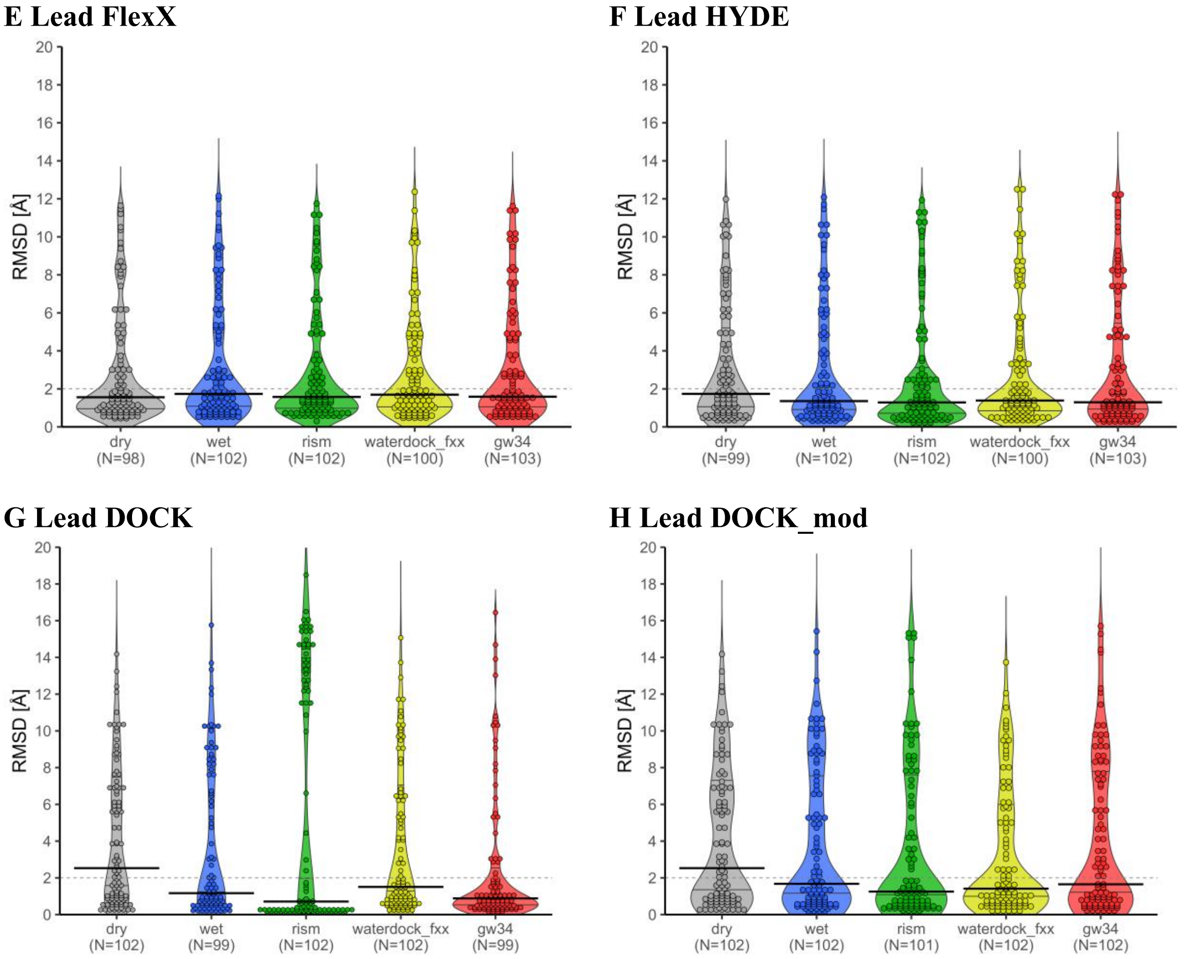

Figure S3 continued: Violine plots of RMSD distributions for water model dry (grey), wet (blue), rism (green), waterdock_fxx (yellow) and gw34 (red) using **A**) FlexX, **B**) HYDE, **C**) DOCK and **D**) DOCK_mod as docking programs for fragment re-docking of the Frag2Lead dataset and **E**) FlexX, **F**) HYDE, **G**) DOCK and **H**) DOCK_mod as docking programs for lead re-docking of the Frag2Lead dataset. The bold horizontal bars indicate the median and the thin horizontal lines within the violine body represent the first and third quartile.

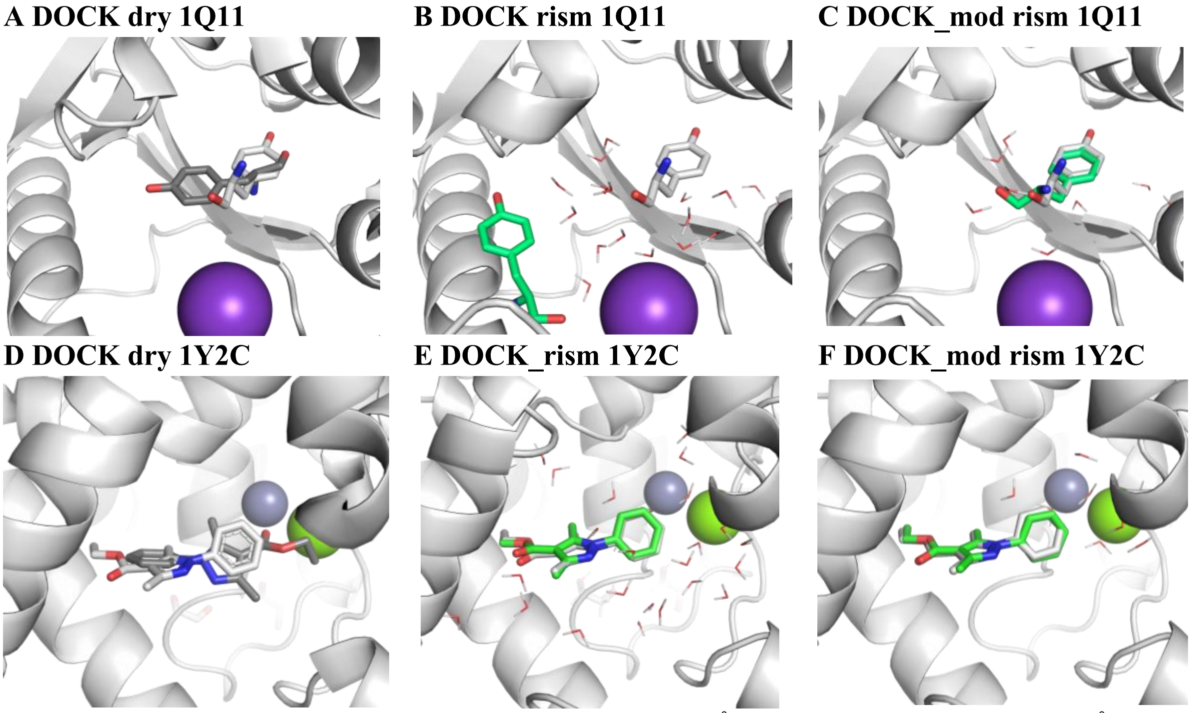

Figure S4: Comparison of **A**) DOCK *dry* (re-docking RMSD = 5.3 Å), **B**) DOCK *rism* (RMSD = 10.8 Å) and **C**) DOCK_mod *rism* receptors (RMSD = 1.3 Å) and re-docking poses for tyrosyl-tRNA synthetase (PDB-ID: 1Q11[1]) and **D**) DOCK *dry* (re-docking RMSD = 6.9 Å), **E**) DOCK *rism* (RMSD = 0.3 Å) and **F**) DOCK_mod *rism* receptors (RMSD = 0.4 Å) and re-docking poses for the catalytic domain of human phosphodiesterase 4D (PDB-ID: 1Y2C[2]) of the LEADS-Frag dataset. The reference crystal structure pose is shown in light grey for each docking result.

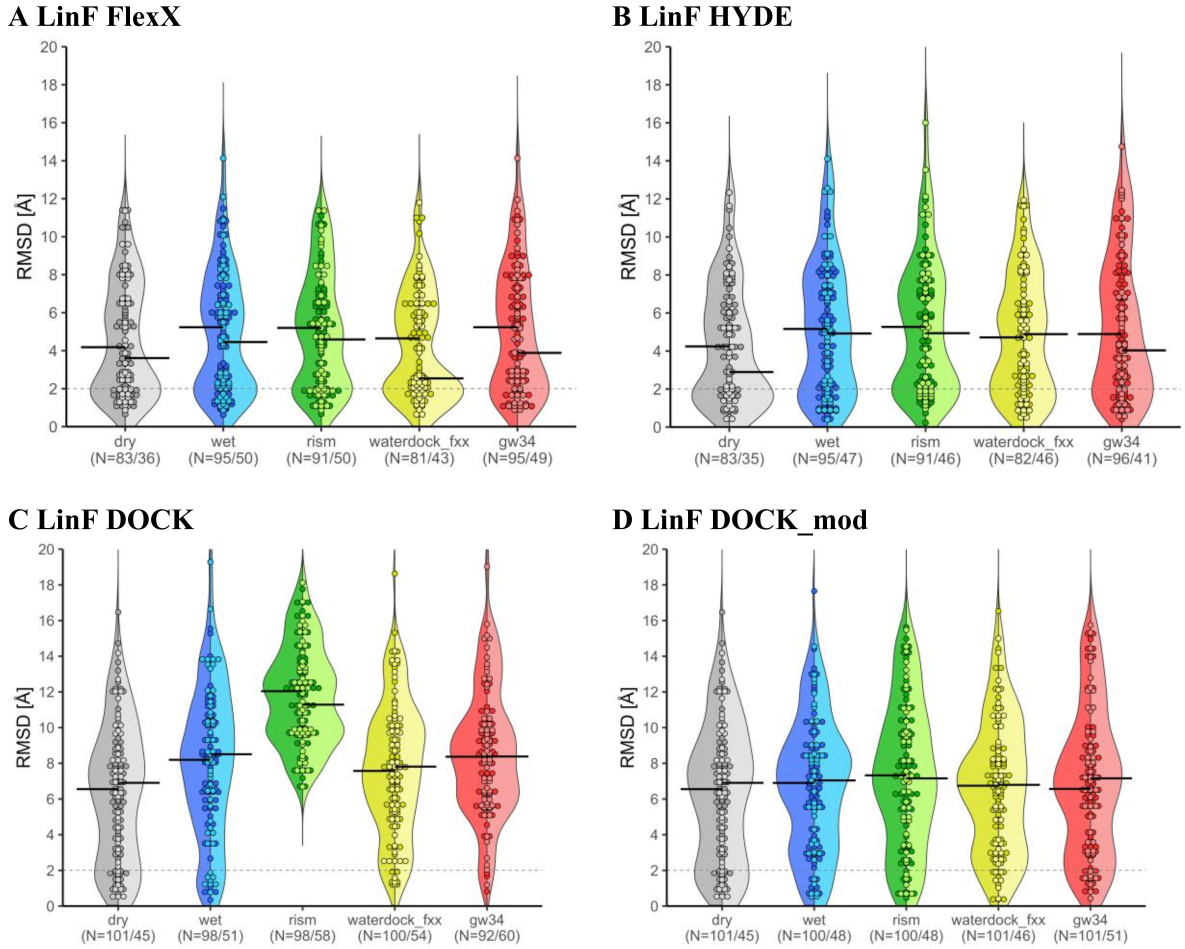

Figure S5: Violine plots of RMSD distributions for water models dry (grey), wet (blue), rism (green), waterdock_fxx (yellow) and gw34 (red) using **A**) FlexX, **B**) HYDE, **C**) DOCK and **D**) DOCK_mod as docking programs for LinF cross-docking of the Frag2Lead dataset. The left side of each violine plot includes all datapoints available, the right side represents cross-docking datapoint for which the corresponding re-docking (for LinF fragment re-docking) was successful (RMSD ≤ 2.0 Å). The bold horizontal lines indicate the median values.

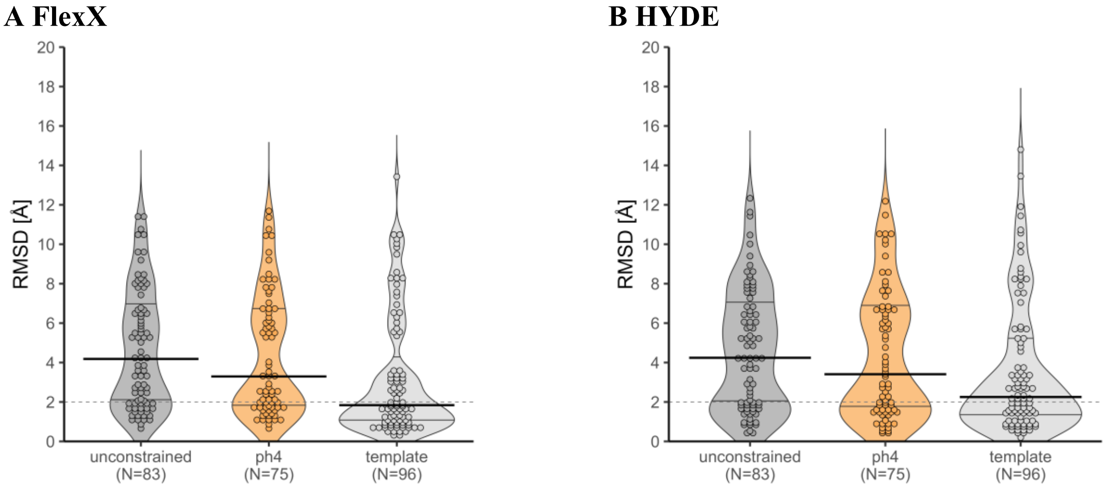

Figure S6: Violine plots of cross-docking RMSD-value distributions for water model *dry* using **A**) FlexX and **B**) HYDE for LinF cross-docking of the Frag2Lead dataset with no constraints (dark grey), a pharmacophore (ph4) constraint (orange) and template docking (light grey). The bold horizontal bars indicate the median.

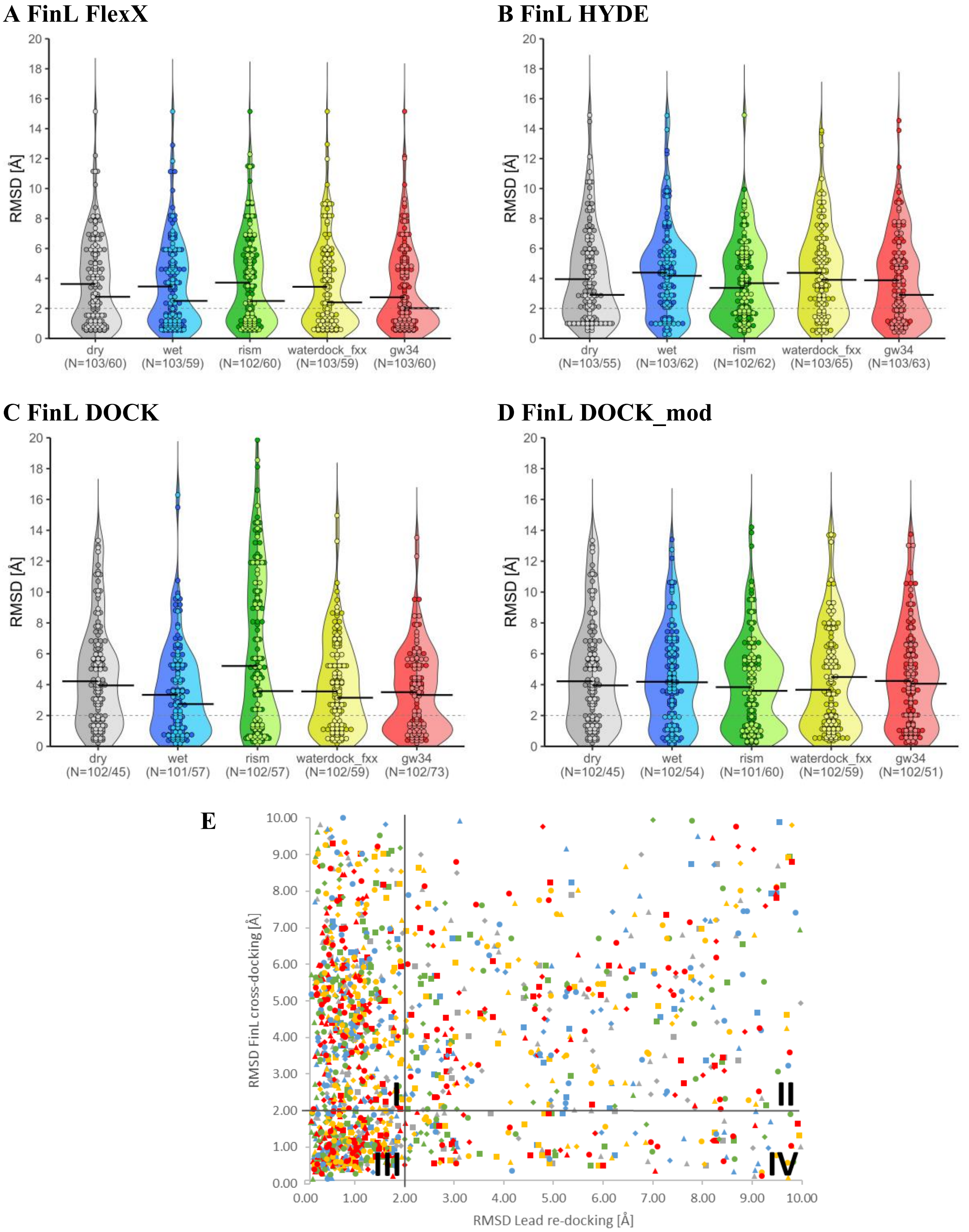

Figure S7: Violine plots of RMSD distributions for water models dry (grey), wet (blue), rism (green), waterdock_fxx (yellow) and gw34 (red) using **A**) FlexX, **B**) HYDE, **C**) DOCK and **D**) DOCK_mod as docking programs for FinL cross-docking of the Frag2Lead dataset. The left side of each violine plot includes all datapoints available, the right side represents cross-docking datapoint for which the corresponding re-docking (for FinL lead-re-docking) was successful (RMSD ≤ 2.0 Å). The bold horizontal lines indicate the median values. **E**) Fragment-in-Lead (FinL) cross-docking RMSD (in Å) plotted against the corresponding lead re-docking RMSD (in Å) for all docking software-solvent model combinations (with RMSD cut-off at 10 Å). Datapoints of FlexX appear in squares, HYDE in rhombi, DOCK in triangles and DOCK_mod in circles for dry (grey), wet (blue), rism (green), waterdock_fxx (yellow) and gw34 (red). Lines at 2.0 Å divide the datapoints in four sectors for the predictive value of re-docking for cross-docking. I: re-docking RMSD ≤2.0 Å + cross-docking RMSD >2.0 Å (false positive). II: re-docking RMSD >2.0 Å + cross-docking RMSD >2.0 Å (true negative). III: re-docking RMSD ≤2.0 Å + cross-docking RMSD ≤2.0 Å (true positive). IV: re-docking RMSD >2.0 Å + cross-docking RMSD ≤2.0 Å (false negative).

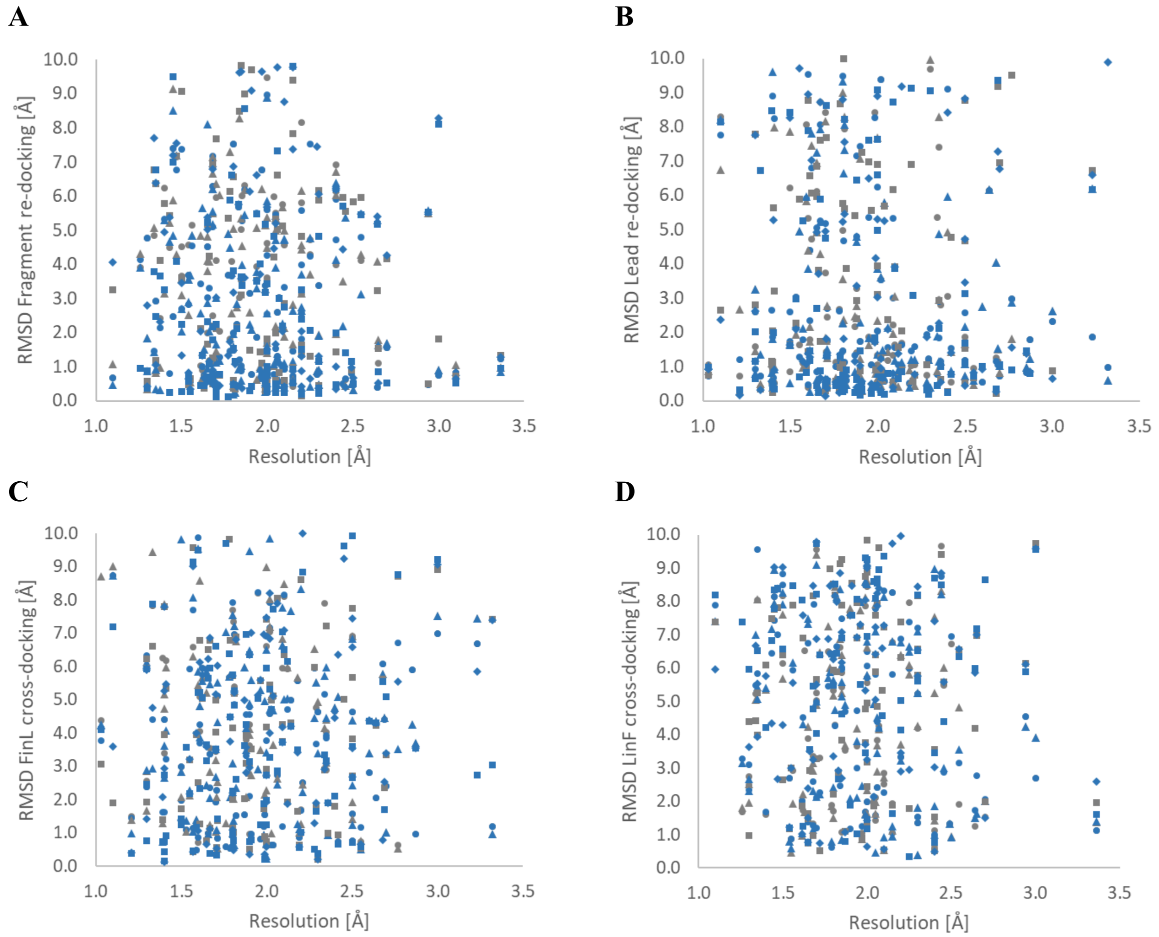

Figure S8: Docking RMSD in Å plotted against the resolution of the corresponding crystal structure in Å (with RMSD cut-off at 10 Å). **A**) fragment re-docking RMSD against fragment crystal structure resolution, **B**) lead re-docking RMSD against lead crystal structure resolution, **C**) Fragment-in-Lead (FinL) cross-docking RMSD against lead crystal structure resolution, **D**) Lead-in-Fragment (LinF) cross-docking RMSD against fragment crystal structure resolution. Datapoints in grey represent *dry* docking setups and blue *wet* docking. Circles correspond to FlexX dockings, triangles to HYDE, squares to DOCK and to rhombus to DOCK_mod.

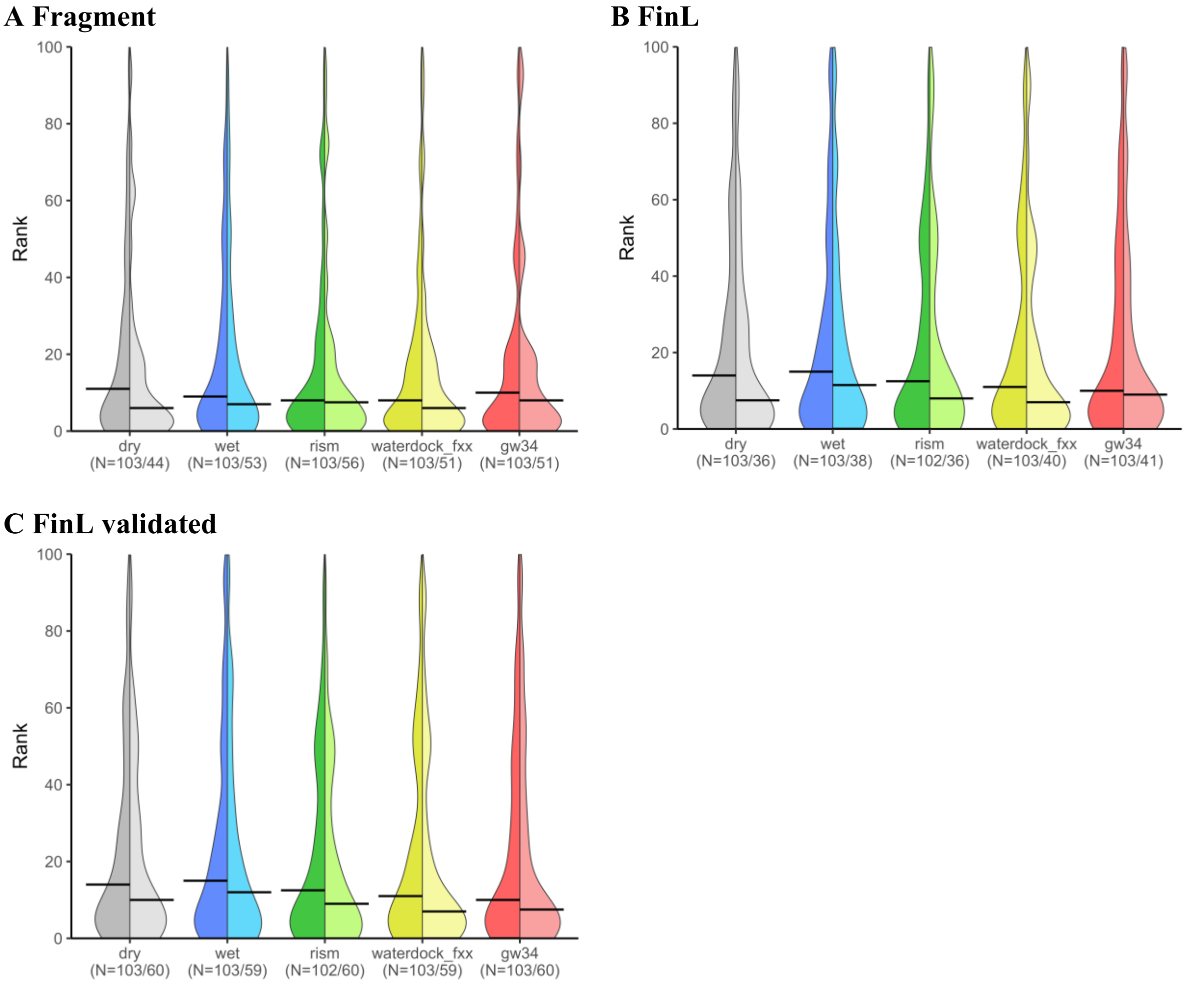

Figure S9: Violine plots of ranking fragment binders from the Frag2Lead dataset against F2X decoys using FlexX for water model *dry* (grey), *wet* (blue), *rism* (green), *waterdock_fxx* (yellow) and *gw34* (red). The left half of the violines includes all Frag2Lead target entries of the particular docking. For **A)** Fragment re-dockings with right half only those with right pose of the fragment re-docking (RMSDs ≤2.0 Å), **B**) FinL cross-dockings with the right half of the violins only those with a correctly predicted pose of the FinL cross-docking (RMSDs ≤2.0 Å), and **C** FinL cross-dockings with right half only those with right pose of the lead re-docking (RMSDs ≤2.0 Å). The number of data points is written under the solvent label. The bold horizontal lines indicate the median.

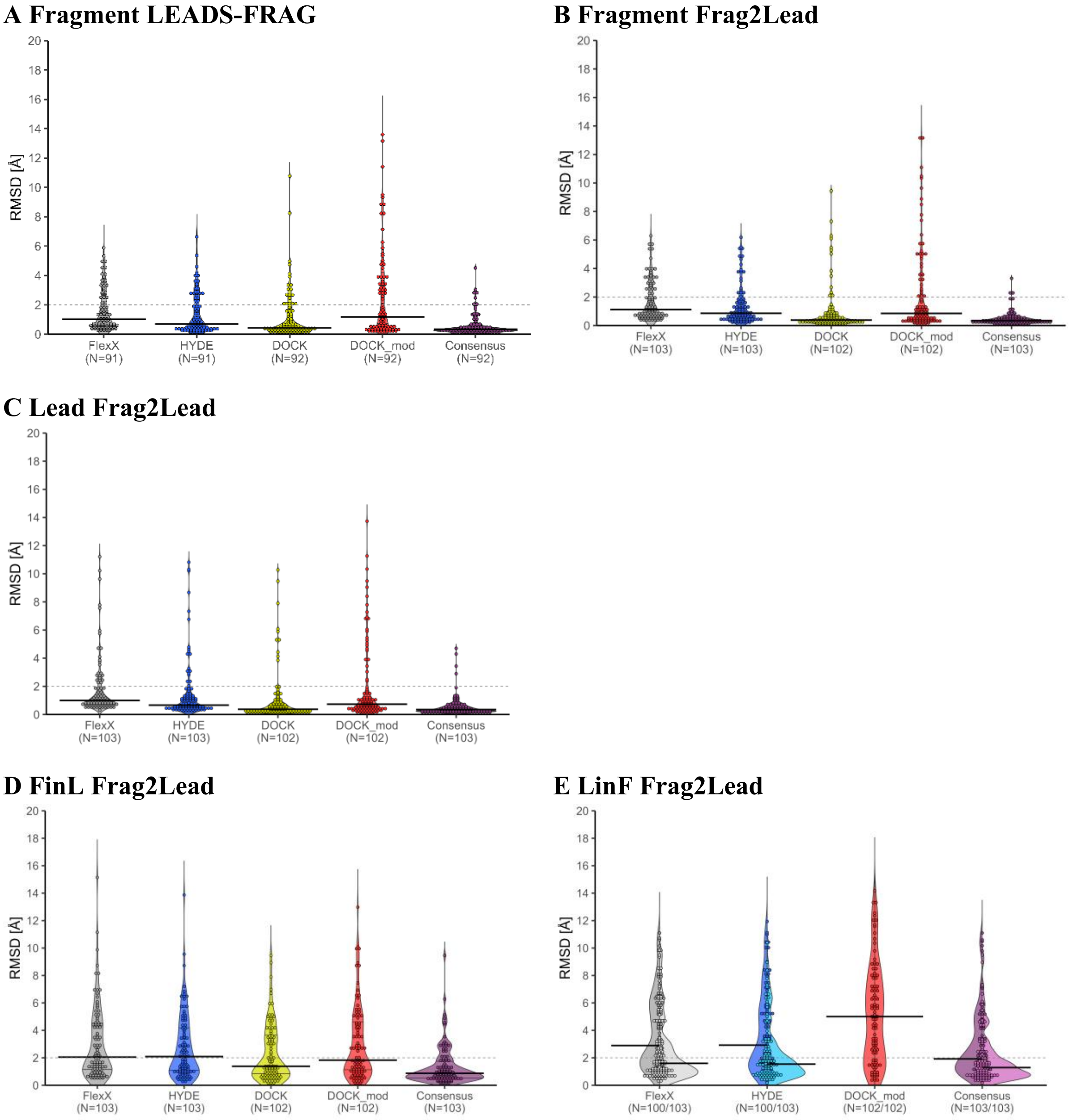

Figure S10: Violine plots of "pick-best" consensus water models RMSD distributions per docking software FlexX (grey), HYDE (blue), DOCK (yellow) and DOCK_mod (red) and consensus model across all software (purple) for **A**) Fragment re-docking of the LEADS-FRAG dataset (to be compared with Figure S2), **B**) Fragment re-docking of the Frag2Lead dataset (to be compared with Figure S3A–D), **C**) Lead re-docking of the Frag2Lead dataset (to be compared with Figure S3E–H), **D**) FinL cross-docking of the Frag2Lead dataset (to be compared with Figure S7) and **E**) LinF cross-docking of the Frag2Lead dataset For E: The left side of each violine plot implies cross-dockings without template docking, the right side includes template cross-docking for FlexX and HYDE (to be compared with Figure S5 and Figure 4). The bold horizontal bars indicate the median.

A Fragment re-docking (LEADS-FRAG dataset)

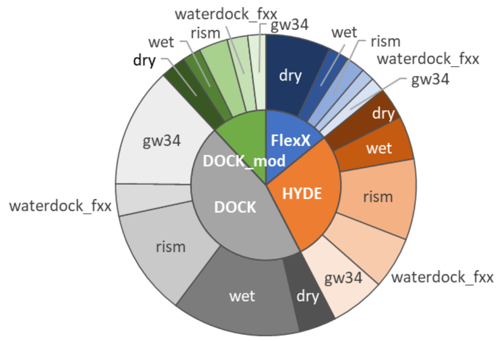

B Fragment re-docking (Frag2Lead dataset)

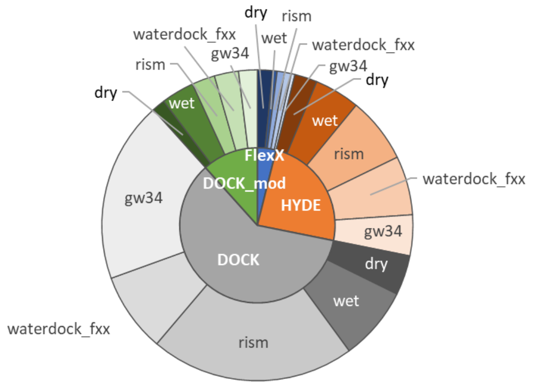

C Lead re-docking (Frag2Lead dataset)

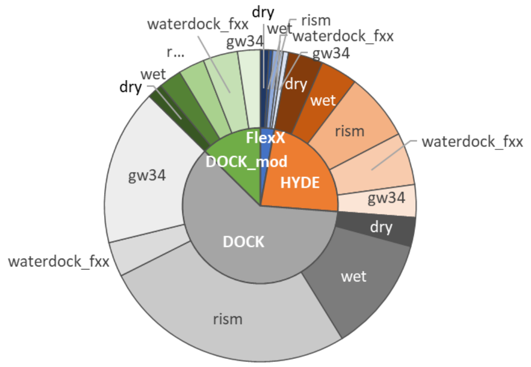

D FinL cross-docking (Frag2Lead dataset)

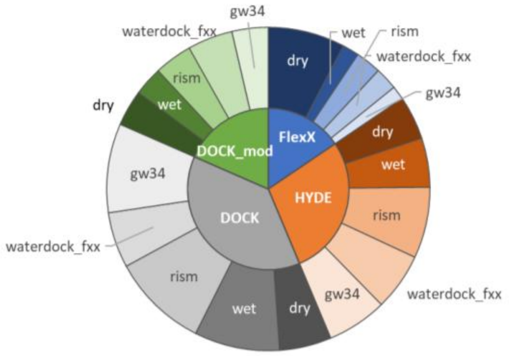

E LinF cross-docking (Frag2Lead dataset)

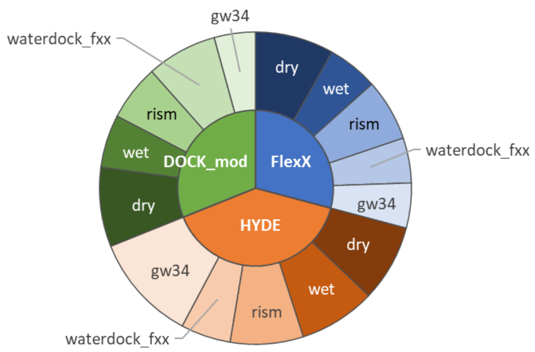

F LinF cross-docking (Frag2Lead dataset) with template

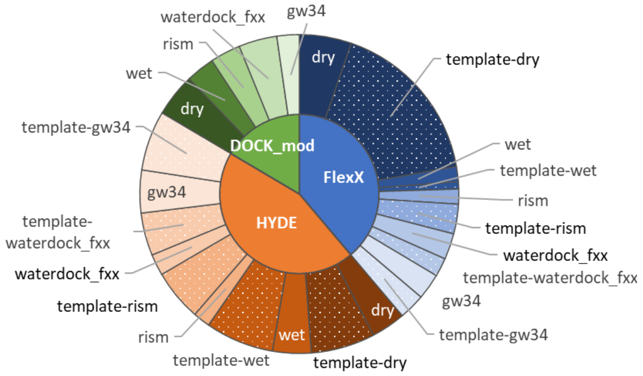

Figure S11: Nested pie charts showing detailed composition of the "pick-best" consensus model over water models and docking software for **A**) fragment re-dockings of the LEADS-FRAG dataset, **B**) fragment re-docking **C**) lead re-docking, **D**) FinL cross-docking, **E**) LinF cross-docking without template and **F**) LinF cross-docking including template docking for FlexX and HYDE of the Frag2Lead dataset.

Table S5: Success rate (number of datapoints), average and median of the cross-docking RMSD values when picking the best and successful (RMSD ≤ 2.0 Å) re-docking water model per entry as water model selection criterium for the cross-dockings (for FinL lead-re-docking and LinF fragment re-docking). For LinF additional differentiation between inclusion and exclusion of the template docking method is shown. Consensus model over water models and docking software is written in bold.

Docking category	Software	Success rate	Average [Å]	Median [Å]
**Fragment-in-Lead**	FlexX	41% (79)	3.6	2.7
	HYDE	29% (84)	4.2	3.1
	DOCK	38% (90)	4.8	3.6
	DOCK_mod	30% (76)	4.5	4.4
	**Consensus**	**35% (96)**	**4.8**	**3.7**
**Lead-in-Fragment**	FlexX	25% (65)	4.4	3.1
	FlexX+template	51% (65)	3.9	1.7
	HYDE	18% (75)	5.4	5.1
	HYDE+template	42% (75)	3.4	2.2
	DOCK_mod	10% (67)	7.0	7.0
	**Consensus**	**17% (90)**	**6.2**	**5.7**
	**+template**	**24% (87)**	**5.6**	**5.0**

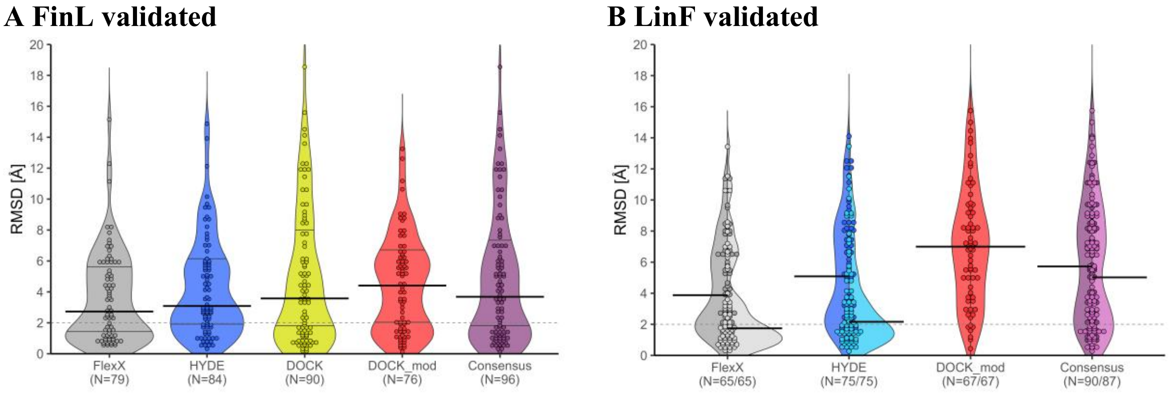

Figure S12: Violine plots of "pick-best" re-docking consensus solvent model for validated cross-docking RMSD distributions (=with right pose of the corresponding re-docking (RMSDs ≤2.0 Å), for FinL lead re-docking and LinF fragment re-docking) using FlexX (grey), HYDE (blue), DOCK (yellow) and DOCK_mod (red) and consensus model across all docking softwares (purple) for **A**) FinL cross-docking, **B**) LinF cross-docking. For B: The left side of each violine plot implies LinF cross-dockings without template docking, the right side includes template cross-docking for FlexX and HYDE. The bold horizontal bars indicate the median.

2. Frag2Lead Dataset: Figure S13, Table S6

2015-01	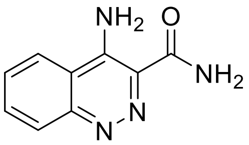	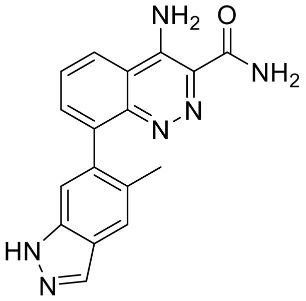
	4ZLY	4Z3V
2015-02	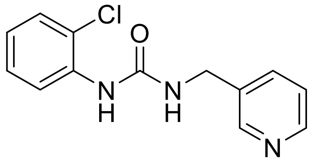	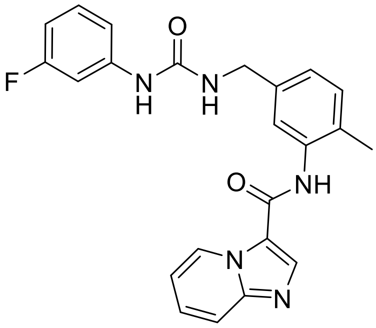
	5BVK	5BVN
2015-03	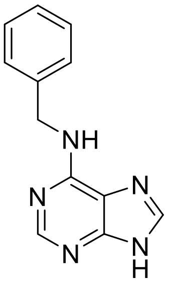	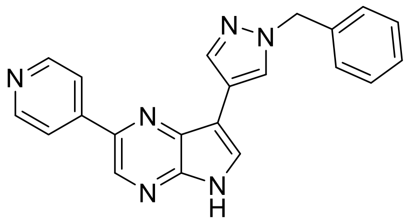
	4QP1	4QPA
2015-04	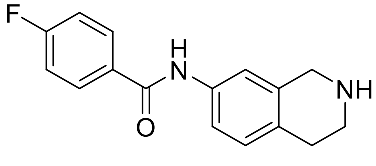	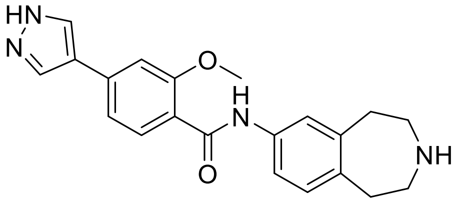
	4UMR	4D2V
2015-05	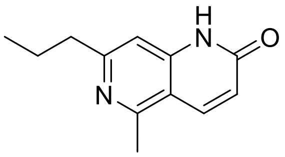	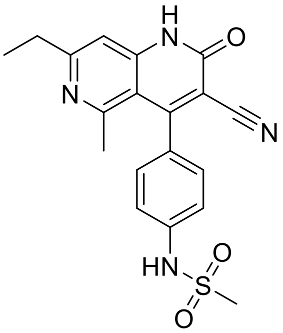
	4UNP	4UNS

Figure S13: Lewis structures of the ligands for all entries in the Frag2Lead dataset. Left structure is fragment and right structure lead compound derived from the paper series *Fragment-to-Lead Medicinal Chemistry Publications*.

2015-06	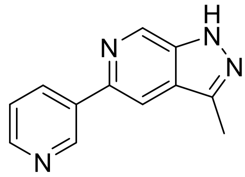	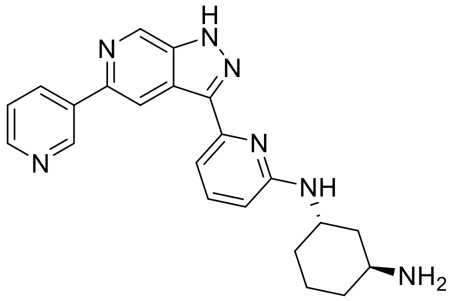
	5DHJ	5DIA
2015-07	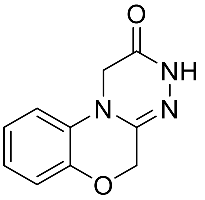	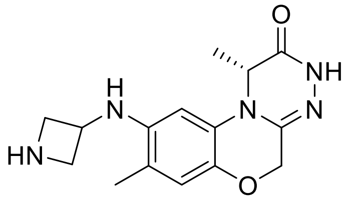
	4Q9S	4Q9Z
2015-08	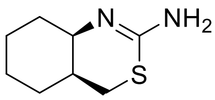	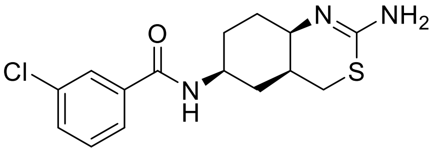
	4ZSM	4ZSP
2015-09	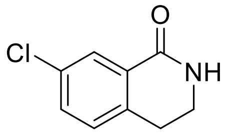	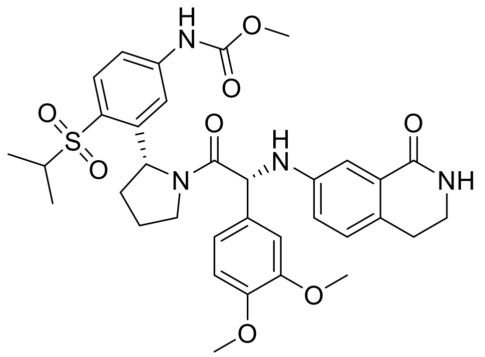
	4X8T	4X8V
2015-10	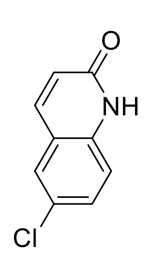	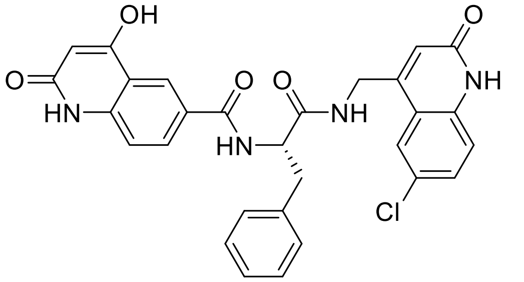
	4CR5	4CRF
2015-11	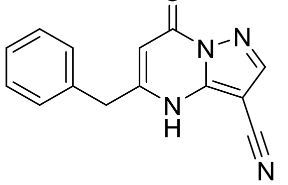	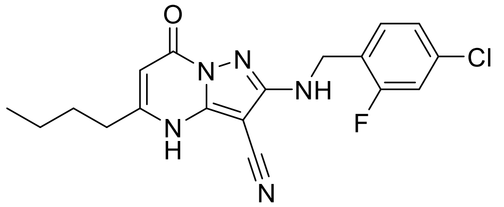
	5BWR	5BWX

Figure S13 continued: Lewis structures of the ligands for all entries in the Frag2Lead dataset. Left structure is fragment and right structure lead compound derived from the paper series *Fragment-to-Lead Medicinal Chemistry Publications*.[3–8]

2015-12	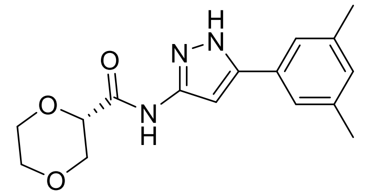	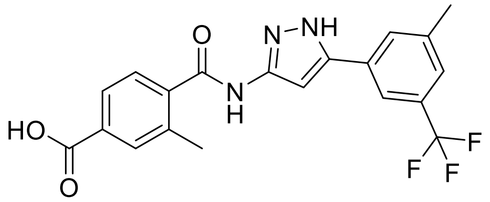
	5BOD	5BOC
2015-13	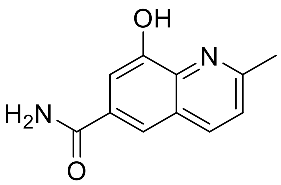	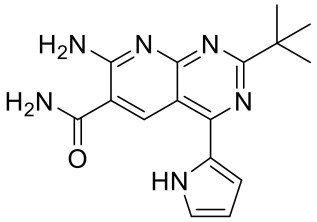
	4UCR	4UCO
2015-14	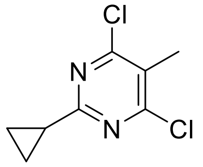	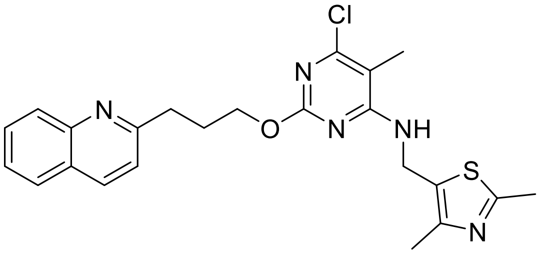
	5C1W	5C2H
2015-15	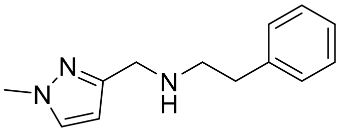	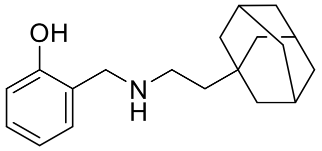
	4Y2J	4Y2X
2015-16	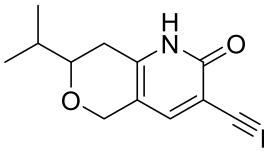	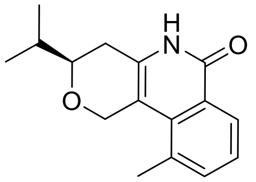
	5C5R	5C5Q
2015-17	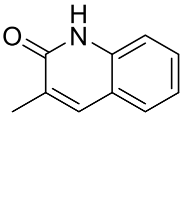	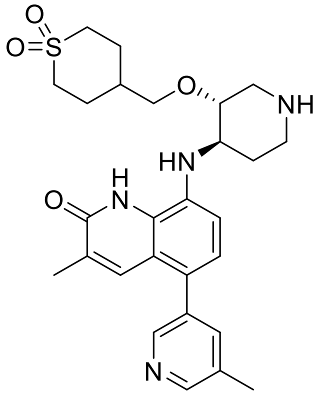
	5A5O	5A83

Figure S13 continued: Lewis structures of the ligands for all entries in the Frag2Lead dataset. Left structure is fragment and right structure lead compound derived from the paper series *Fragment-to-Lead Medicinal Chemistry Publications*.[3–8]

2015-18	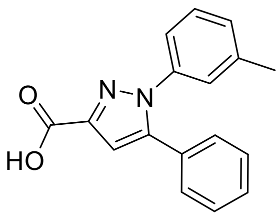	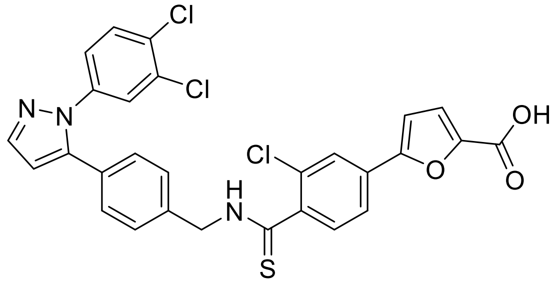
	4LUO	4LWC
2015-19	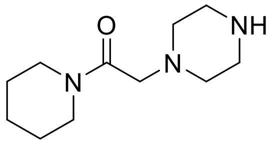	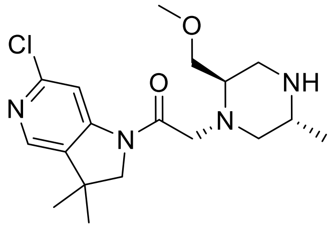
	5C3H	5C84
2016-01	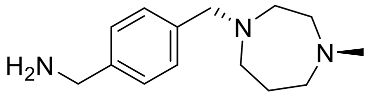	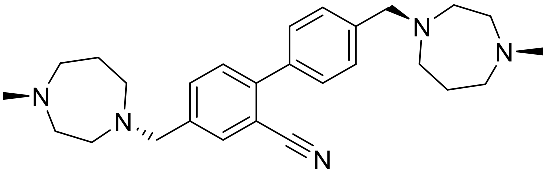
	5EQE	5EQY
2016-02	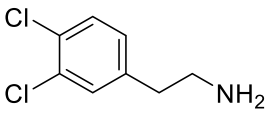	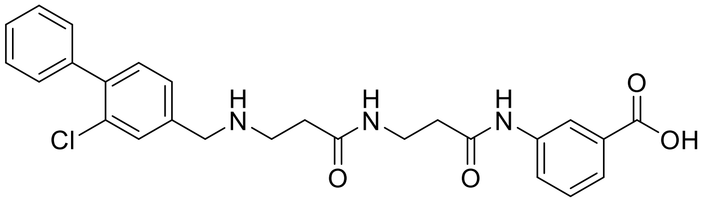
	5CLP	5CU3
2016-03	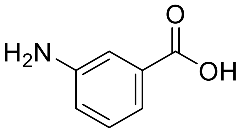	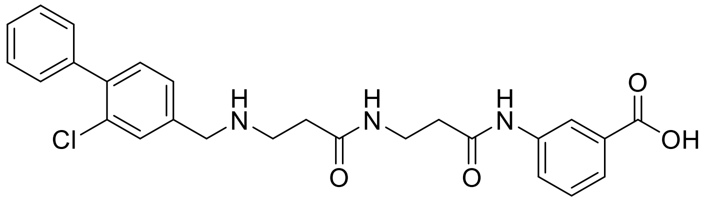
	5CSV	5CU3
2016-04	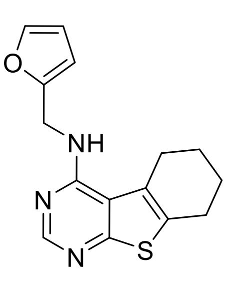	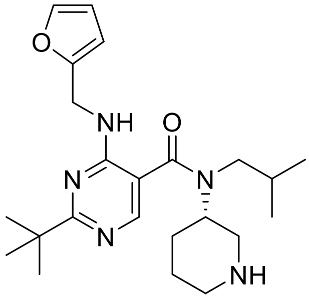
	5SY3	5KOQ

Figure S13 continued: Lewis structures of the ligands for all entries in the Frag2Lead dataset. Left structure is fragment and right structure lead compound derived from the paper series *Fragment-to-Lead Medicinal Chemistry Publications*.[3–8]

2016-05	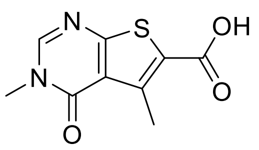	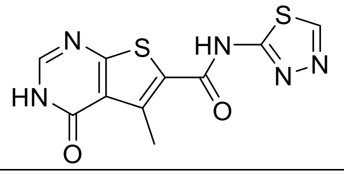
	5I5V	5I5X
2016-06	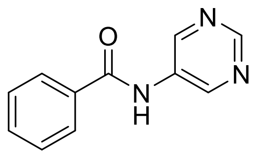	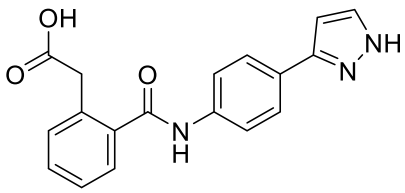
	5I5W	5I60
2016-07	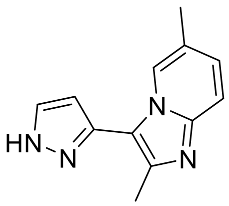	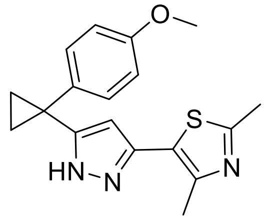
	5K03	5K0L
2016-08	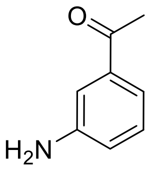	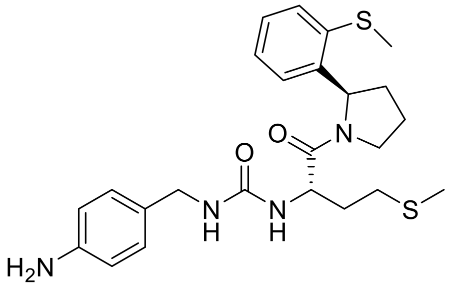
	3R59	4J5C
2016-09	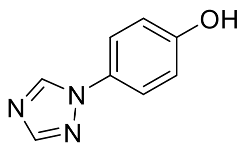	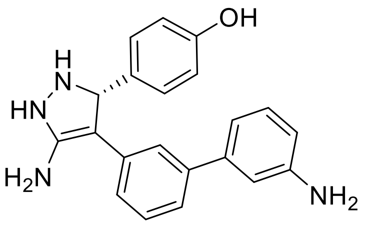
	4G47	5IBE
2016-10	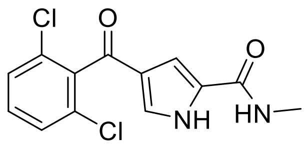	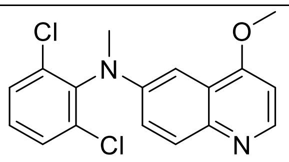
	5DTM	5DTR
2016-11	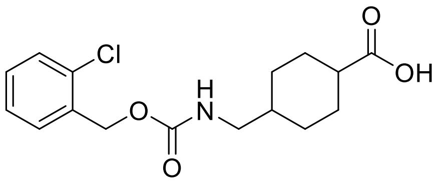	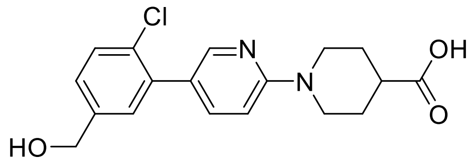
	4Z8D	5BQS

Figure S13 continued: Lewis structures of the ligands for all entries in the Frag2Lead dataset. Left structure is fragment and right structure lead compound derived from the paper *series Fragment-to-Lead Medicinal Chemistry Publications*.[3–8]

2016-12	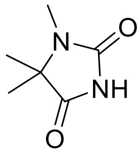	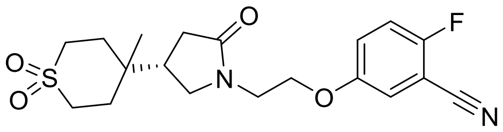
	5JAH	5LZ9
2016-13	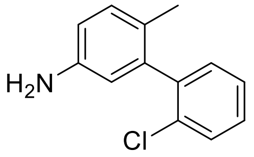	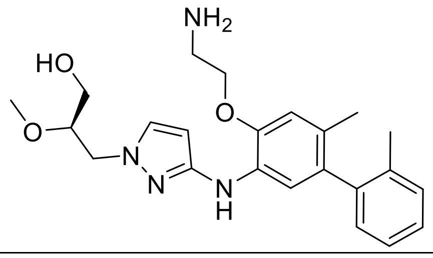
	5JAO	5JAU
2016-14	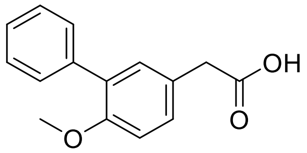	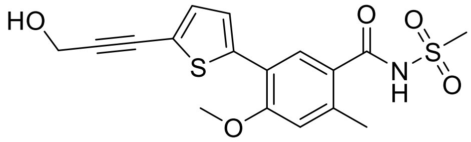
	5F3T	5HMZ
2016-15	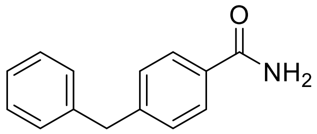	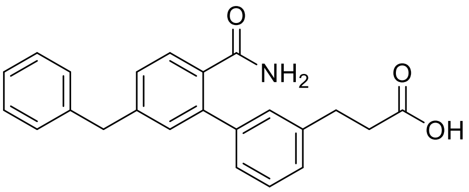
	5G3M	5G3N
2016-16	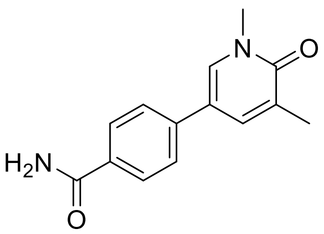	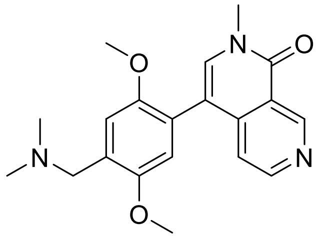
	5F25	5F1H
2016-17	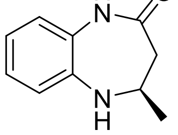	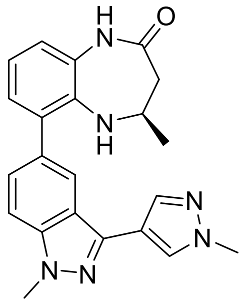
	4YK0	5I8G

Figure S13 continued: Lewis structures of the ligands for all entries in the Frag2Lead dataset. Left structure is fragment and right structure lead compound derived from the paper series *Fragment-to-Lead Medicinal Chemistry Publications*.[3–8]

2016-18	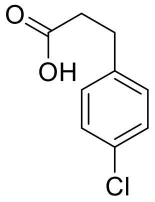	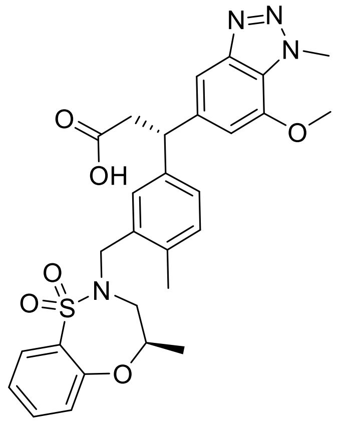
	5FNQ	5FNU
2016-19	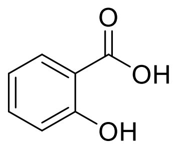	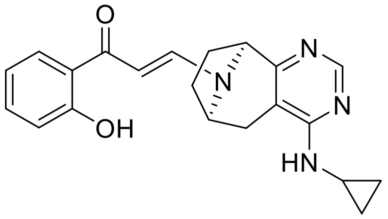
	4Y03	5DKH
2016-20	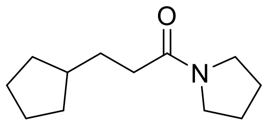	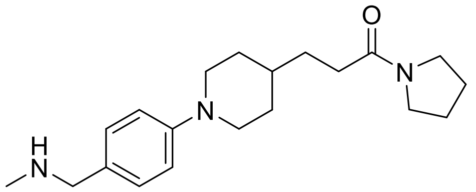
	5F1J	5EYR
2016-21	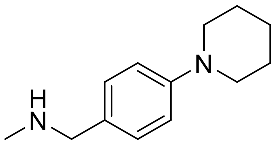	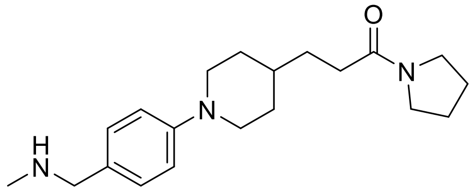
	5F27	5EYR
2017-01	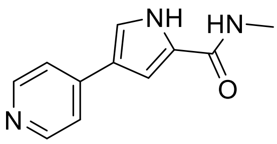	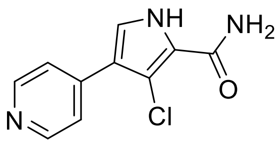
	5XQX	5XS2
2017-02	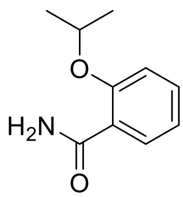	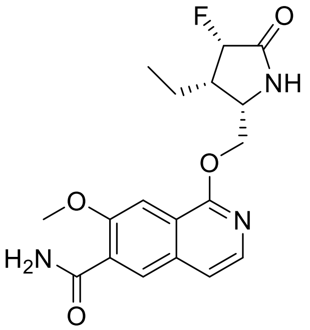
	5UIQ	5UIU

Figure S13 continued: Lewis structures of the ligands for all entries in the Frag2Lead dataset. Left structure is fragment and right structure lead compound derived from the paper series *Fragment-to-Lead Medicinal Chemistry Publications*.[3–8]

2017-03	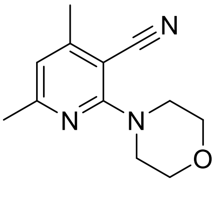	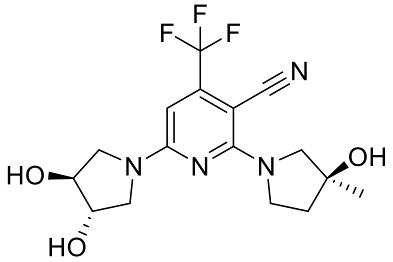
	5WBO	5WBZ
2017-04	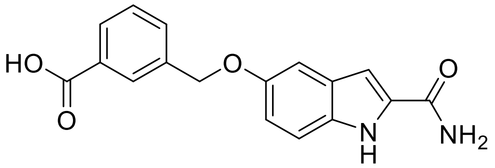	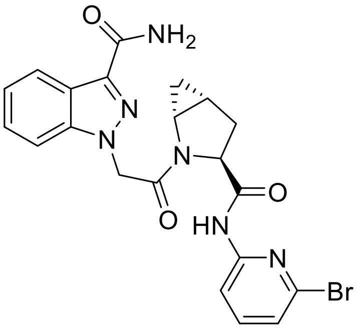
	5FBI	5NB7
2017-05	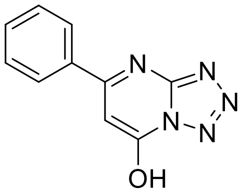	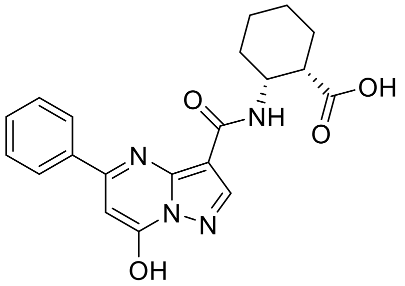
	5V8O	6NAO
2017-06	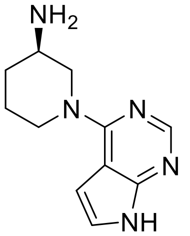	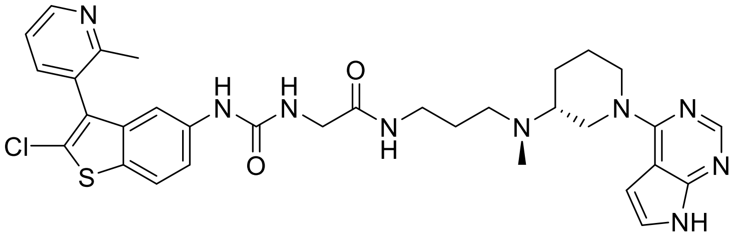
	5MW3	5MW4
2017-07	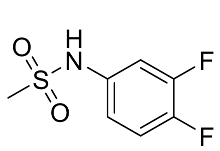	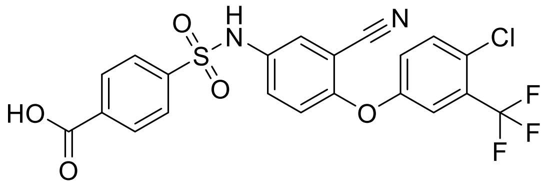
	5YE8	5YEA

Figure S13 continued: Lewis structures of the ligands for all entries in the Frag2Lead dataset. Left structure is fragment and right structure lead compound derived from the paper series *Fragment-to-Lead Medicinal Chemistry Publications*.[3–8]

2017-08	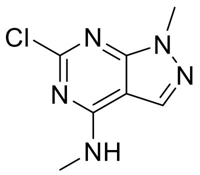	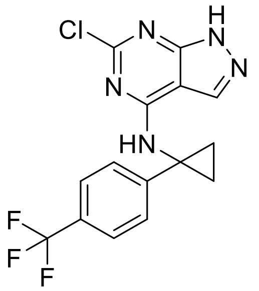
	6B98	6B96
2017-09	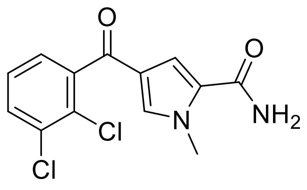	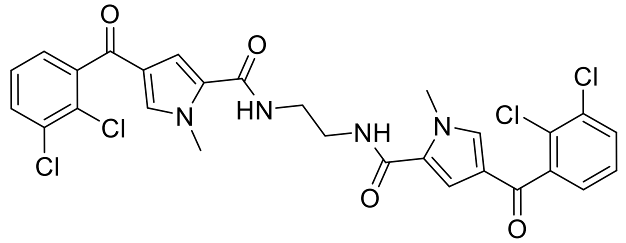
	5X1V	5X1W
2017-10	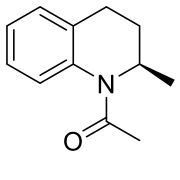	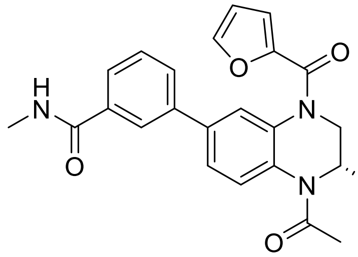
	4A9H	5VOM
2017-11	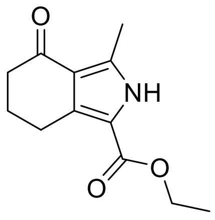	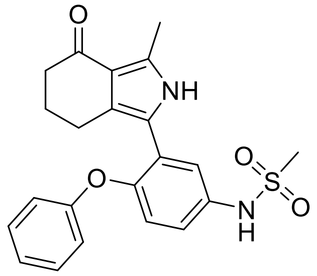
	5UEP	5UER
2017-12	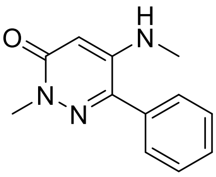	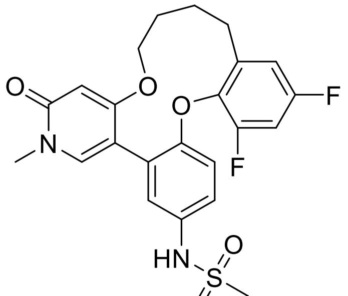
	5uf0	5UEX

Figure S13 continued: Lewis structures of the ligands for all entries in the Frag2Lead dataset. Left structure is fragment and right structure lead compound derived from the paper series *Fragment-to-Lead Medicinal Chemistry Publications*.[3–8]

2017-13	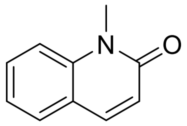	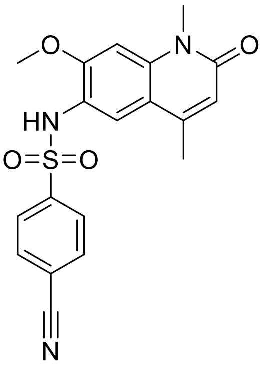
	5T4U	5T4V
2017-14	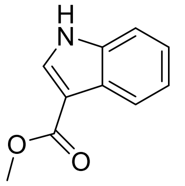	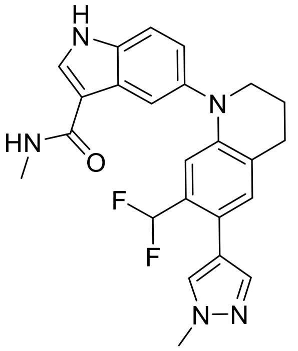
	6AXQ	6AY3
2017-15	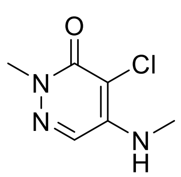	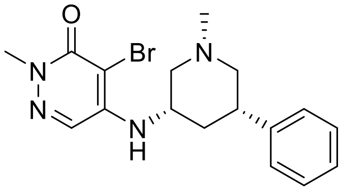
	5MKX	5MLJ
2017-16	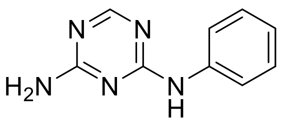	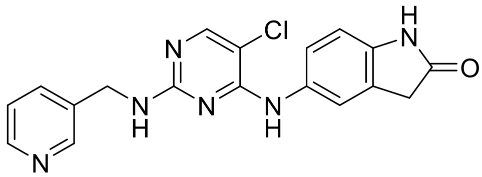
	5X4M	5X4Q
2017-17	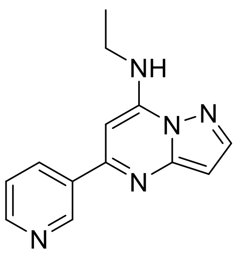	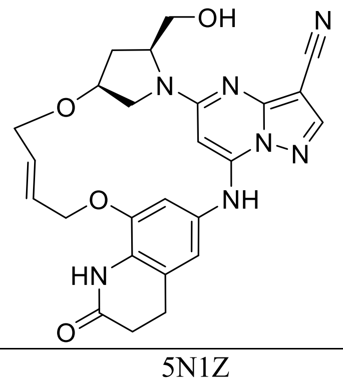
	5N1X	5N1Z

Figure S13 continued: Lewis structures of the ligands for all entries in the Frag2Lead dataset. Left structure is fragment and right structure lead compound derived from the paper series *Fragment-to-Lead Medicinal Chemistry Publications*.[3–8]

Entry	Fragment	Lead
2017-18	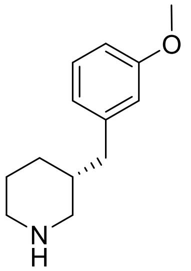	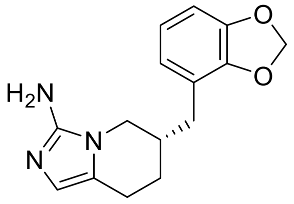
	5U5K	5U62
2018-01	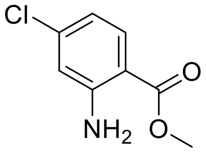	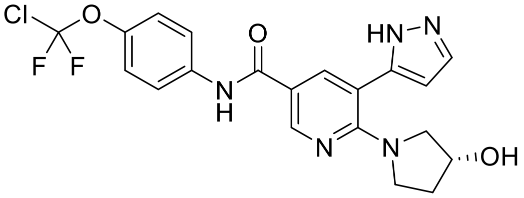
	3MS9	5MO4
2018-02	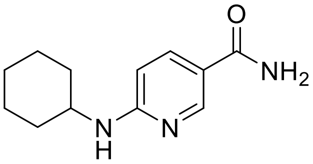	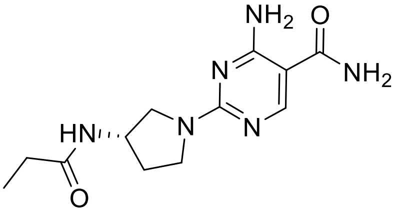
	6DI0	6DI1
2018-03	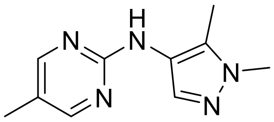	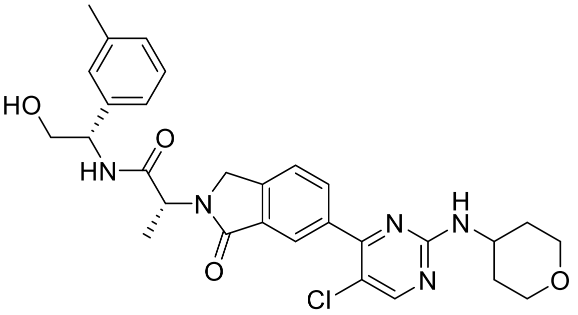
	6G92	6G9N
2018-04	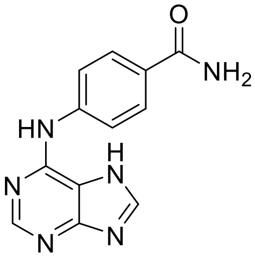	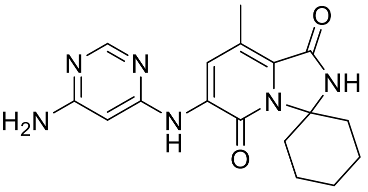
	6CJE	6CK6
2018-05	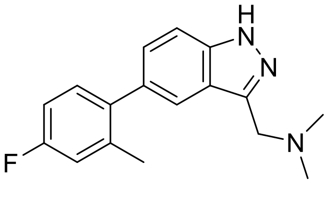	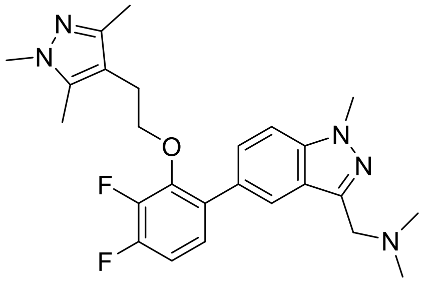
	5O4V	5MU6

Figure S13 continued: Lewis structures of the ligands for all entries in the Frag2Lead dataset. Left structure is fragment and right structure lead compound derived from the paper series *Fragment-to-Lead Medicinal Chemistry Publications.*[3–8]

2018-06	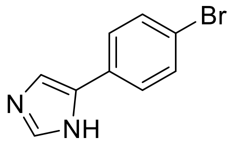	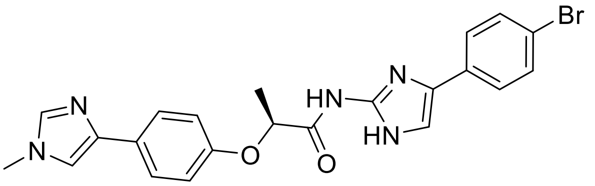
	5OU2	5OU3
2018-07	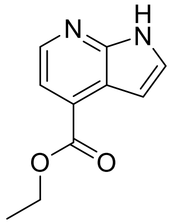	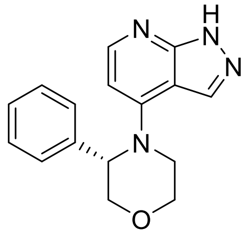
	6F20	6F22
2018-08	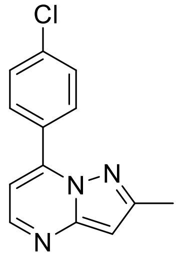	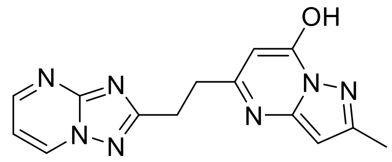
	5XUJ	5XUI
2018-09	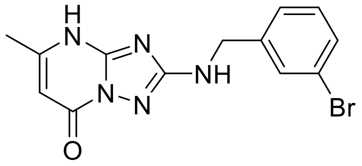	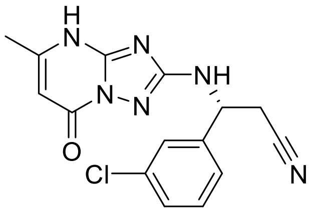
	6CCM	6CCK
2018-10	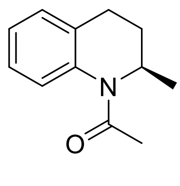	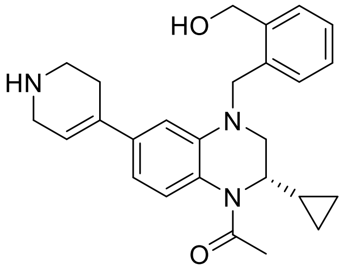
	4A9H	6FFG
2018-11	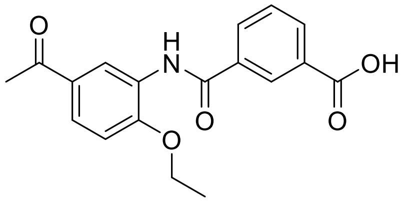	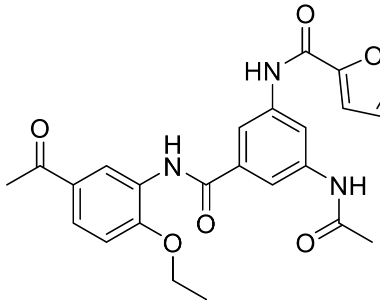
	4TQN	5NLK

Figure S13 continued: Lewis structures of the ligands for all entries in the Frag2Lead dataset. Left structure is fragment and right structure lead compound derived from the paper series *Fragment-to-Lead Medicinal Chemistry Publications*.[3–8]

Entry	Fragment	Lead
2018-12	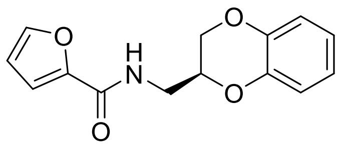	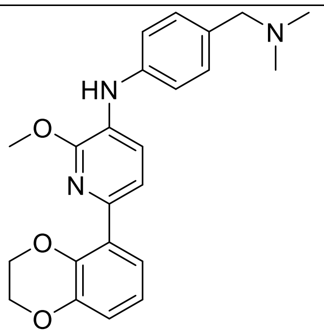
	5OCO	6FA4
2018-13	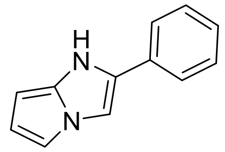	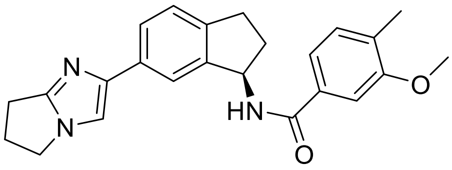
	6D9X	6DAS
2018-14	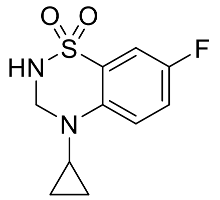	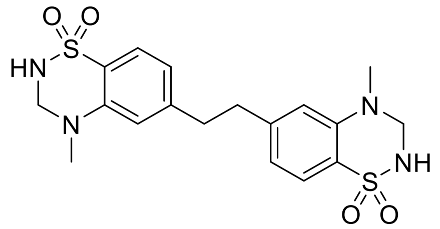
	4N07	6FAZ
2018-15	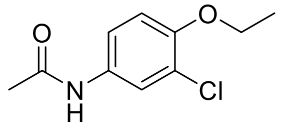	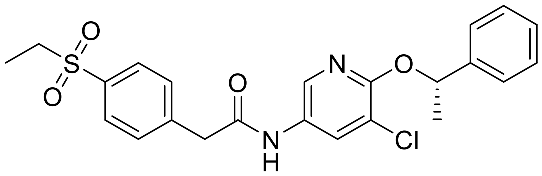
	6FZU	6G07
2019-01	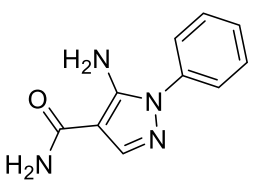	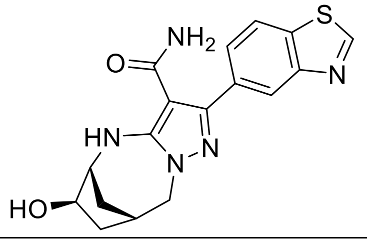
	6SZE	6UL8
2019-02	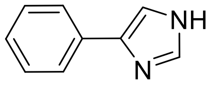	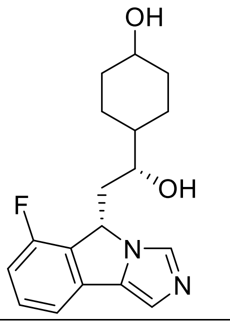
	2D0T	6O3I

Figure S13 continued: Lewis structures of the ligands for all entries in the Frag2Lead dataset. Left structure is fragment and right structure lead compound derived from the paper series *Fragment-to-Lead Medicinal Chemistry Publications*.[3–8]

2019-03	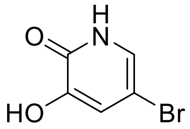	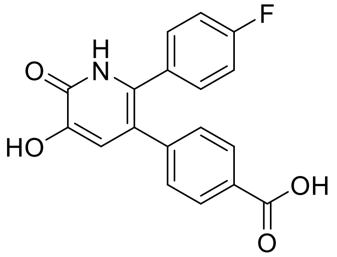
	4MK1	6NEL
2019-04	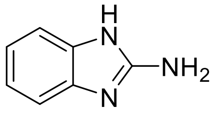	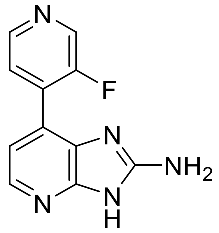
	6EQ5	6EQ7
2019-05	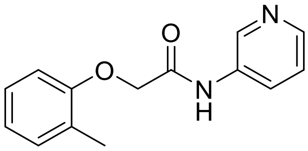	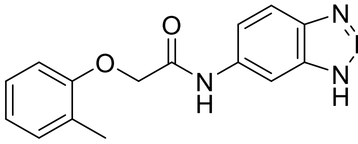
	6R8P	6R8Q
2019-06	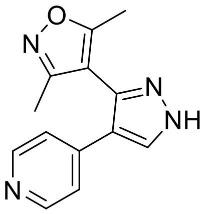	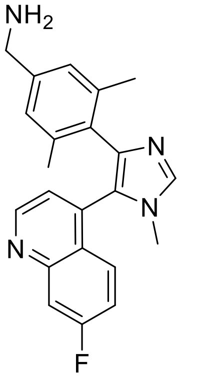
	6G25	6G2O
2019-07	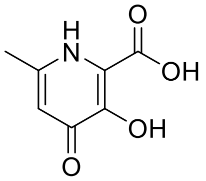	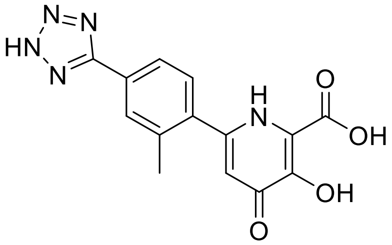
	6E6V	6E6W
2019-08	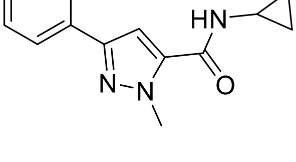	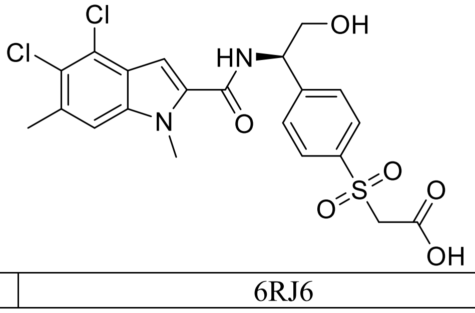
	6RIH	6RJ6

Figure S13 continued: Lewis structures of the ligands for all entries in the Frag2Lead dataset. Left structure is fragment and right structure lead compound derived from the paper series *Fragment-to-Lead Medicinal Chemistry Publications*.[3–8].

2019-09	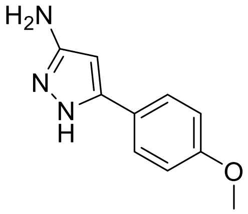	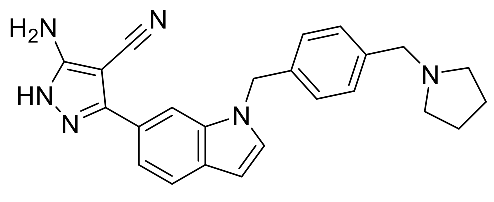
	6QOT	6QR6
2019-10	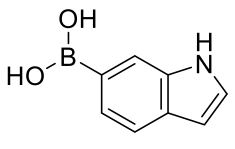	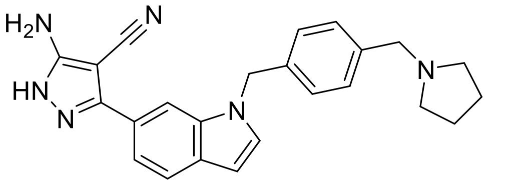
	6QOU	6QR6
2019-11	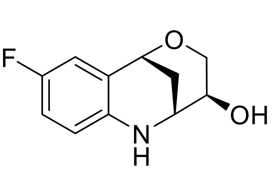	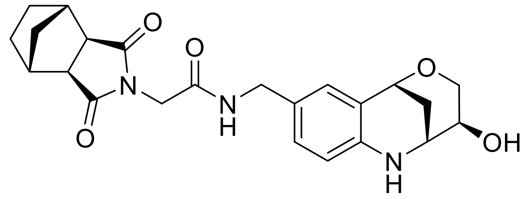
	6R9S	6R8W
2019-12	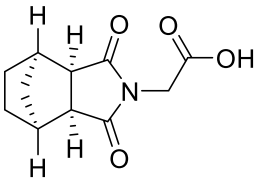	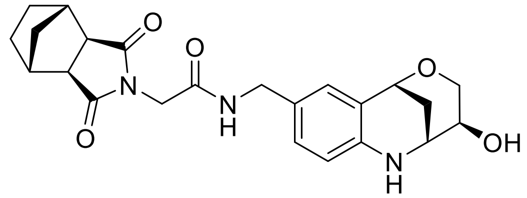
	6RA1	6R8W
2019-13	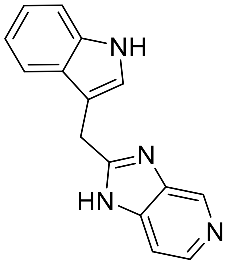	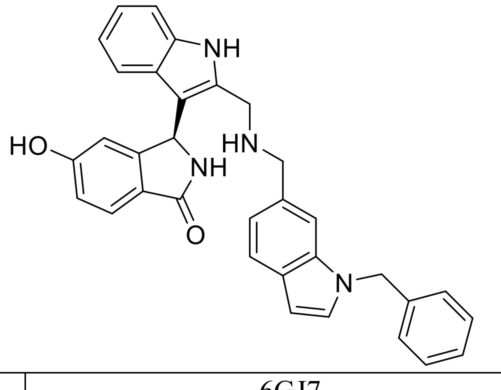
	4EPV	6GJ7
2019-14	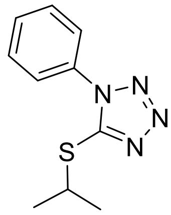	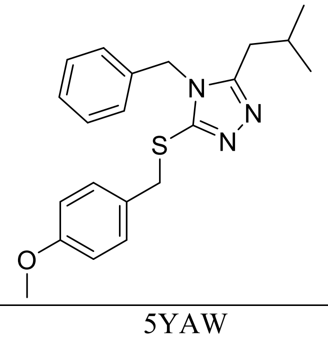
	5YAV	5YAW

Figure S13 continued: Lewis structures of the ligands for all entries in the Frag2Lead dataset. Left structure is fragment and right structure lead compound derived from the paper series *Fragment-to-Lead Medicinal Chemistry Publications*.[3–8]

2019-15	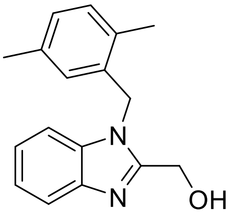	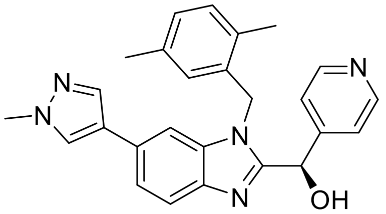
	6OOY	6OP0
2019-16	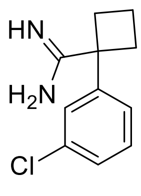	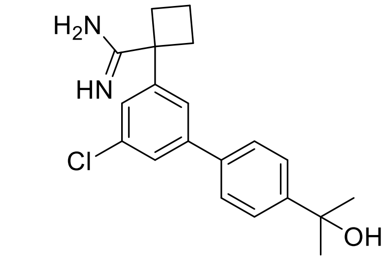
	6NCN	6NCO
2020-01	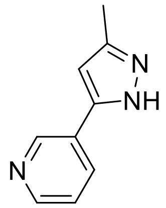	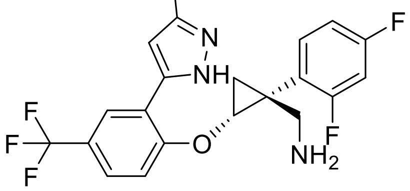
	7JYT	7JY4
2020-02	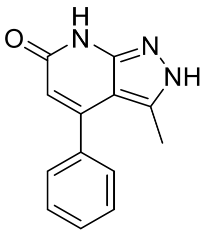	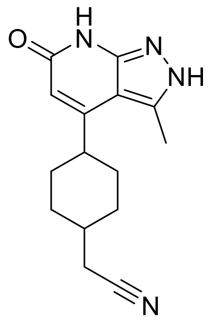
	6TPD	6TPE
2020-03	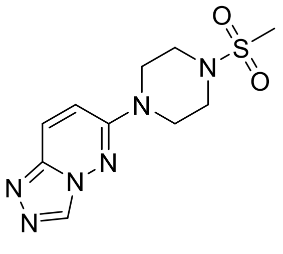	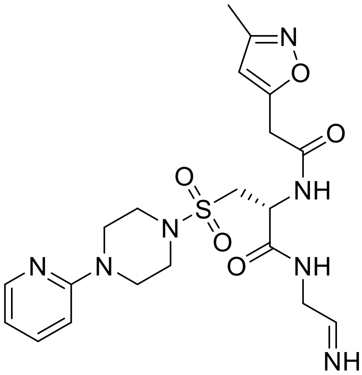
	6YYO	6YYR

Figure S13 continued: Lewis structures of the ligands for all entries in the Frag2Lead dataset. Left structure is fragment and right structure lead compound derived from the paper series *Fragment-to-Lead Medicinal Chemistry Publications*.[3–8]

2020-04	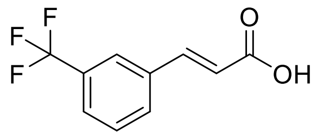	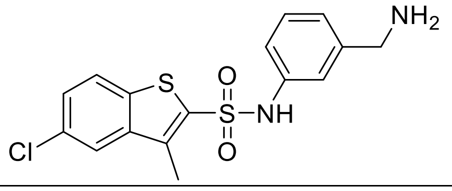
	6SQ5	6SQL
2020-05	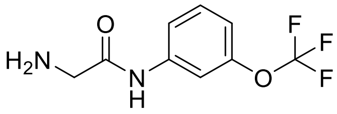	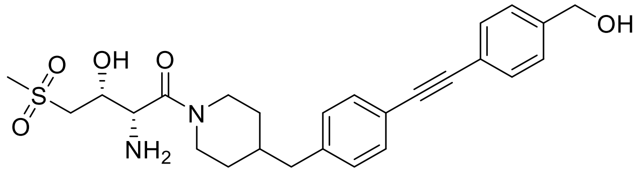
	7CIC	7CI7
2020-06	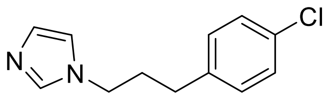	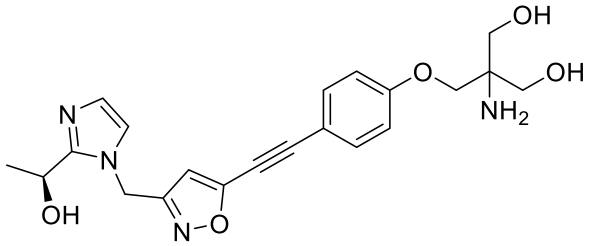
	7CID	7CI9
2020-07	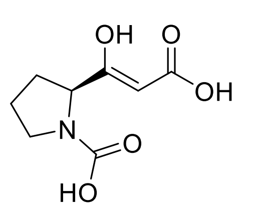	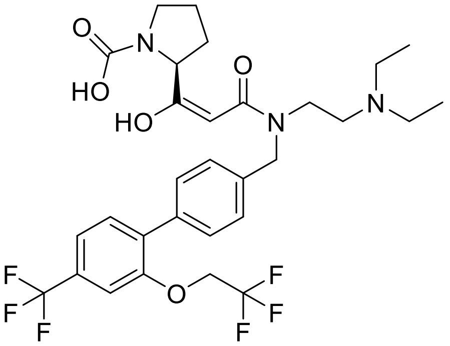
	6M06	6M07
2020-08	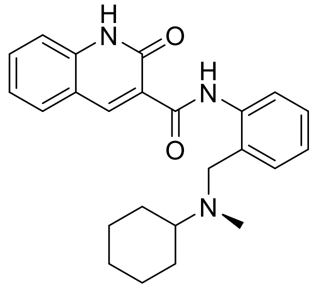	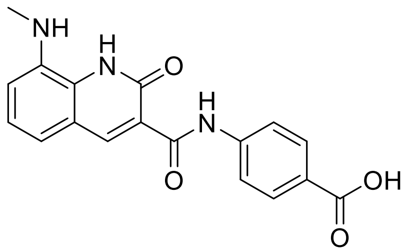
	6KZV	6KZZ
2020-09	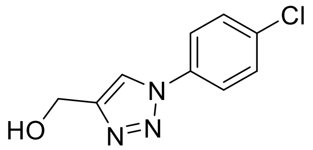	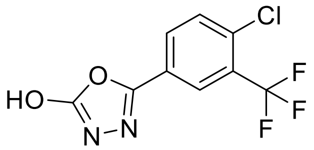
	6ZUV	6ZVL

Figure S13 continued: Lewis structures of the ligands for all entries in the Frag2Lead dataset. Left structure is fragment and right structure lead compound derived from the paper series *Fragment-to-Lead Medicinal Chemistry Publications*.[3–8]

2020-10	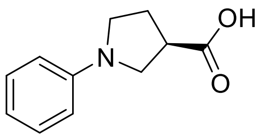	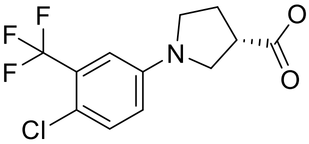
	6YV2	6YSK
2020-11	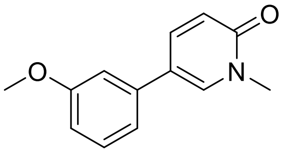	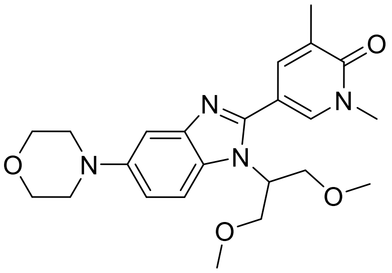
	6TQ1	6TPZ
2020-12	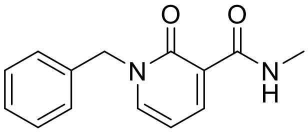	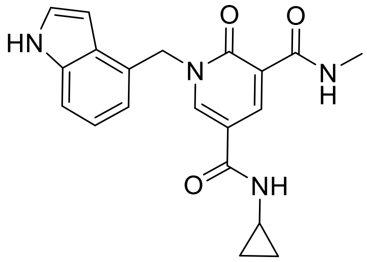
	6ZB0	6ZB2
2020-13	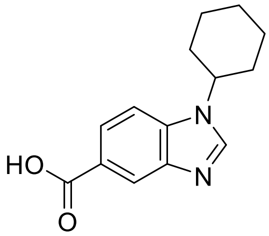	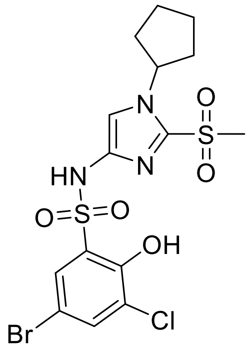
	6UHY	6UOZ
2020-14	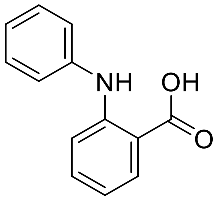	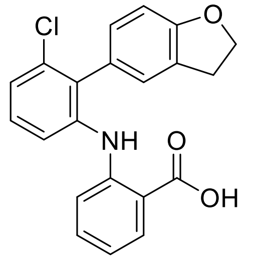
	6LJW	6LJV

Figure S13 continued: Lewis structures of the ligands for all entries in the Frag2Lead dataset. Left structure is fragment and right structure lead compound derived from the paper series *Fragment-to-Lead Medicinal Chemistry Publications.*[3–8]

Table S6: Average values of physicochemical parameters of the used datasets with standard deviations. Raw data can be found in Table S7, Table S8 and Table S10 (XLSX).

Parameter	LEADS-FRAG	Frag2Lead Fragments	F2X	Frag2Lead Leads
Molecular Weight in Da	184.9 ± 52.8	217.1 ± 45.7	183.7 ± 37.1	396.3 ± 88.5
TPSA in $Å^2$	58.3 ± 26.5	51.4 ± 19.4	46.1 ± 15.2	87.6 ± 28.9
Lip_acceptors	3.6 ± 1.6	3.7 ± 1.3	3.2 ± 1.1	6.4 ± 1.9
Lip_donors	2.0 ± 1.6	1.5 ± 1.0	1.3 ± 0.9	2.4 ± 1.4
h_logP	0.8 ± 2.5	1.8 ± 1.1	1.3 ± 0.8	3.1 ± 1.5
logP(o/w)	1.1 ± 1.4	1.7 ± 1.1	1.3 ± 0.8	3.0 ± 1.7
SlogP	1.1 ± 1.5	1.9 ± 1.1	1.5 ± 0.8	3.4 ± 1.6

3. Extended Material and Methods – Implementation of waterdock_fxx: Figure S14

The waterdock_fxx method consists of two parts. First, a water molecule is docked within the binding site using FlexX (flexx-waterdock.py). This can be either performed in presence (holo, Figure S14A) or absence (apo, Figure S14B) of the reference ligand (Figure S14A). If the reference ligand shall be considered as part of the binding site, it is required in the receptor, but binding site definition (-r keyword) must differ. Up to 100 water molecules are docked and filtered in a second step. Using the python script topwater.py with the "cluster" keyword, water molecules are clustered based on their coordinates and the best scoring water molecule per cluster is kept, while water molecules within a user-defined radius between 0.8 and 1.6 Å (1.0 Å default) are removed (Figure S14B). Alternatively, using the "cascade" method, the best scoring water molecule is identified. If not too close to the ligand (apo keyword in step 1, minimal ligand distance between 2.6 and 3.0 Å (3.0 Å default) can be defined by the user), it is kept and all water molecules within a user-defined distance between 2.0 to 3.0 Å (2.8 Å default) are removed (Figure S14C,D). Then, the procedure continues with the next best scoring water molecule. The python scripts flexx-waterdock.py and topwater.py and usage instructions are available in the Supporting Information (PY, ZIP). To run waterdock_fxx, a valid license of FlexX is required.

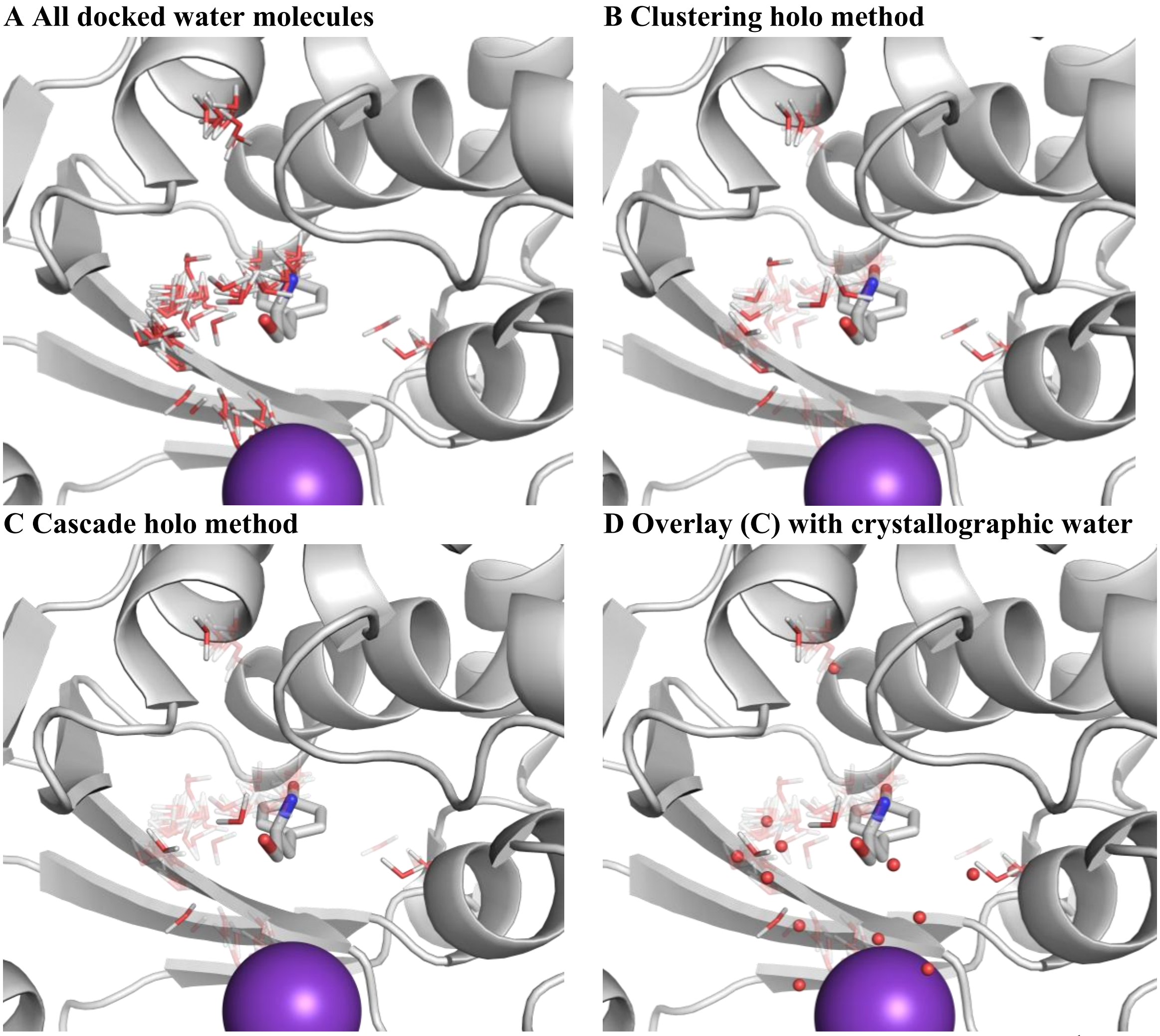

Figure S14: Hydrated complex structures of tyrosyl-tRNA synthetase with its ligand (PDB-ID: 1Q11[1]) using the waterdock_fxx method. **A**) Output with all docked water molecules using flexx-waterdock.py. Filtering of the docked water molecules using topwater.py with **B**) clustering holo method with default settings 3.0 Å ligand distance and 1.0 Å method distance and **C**) cascade holo method with default settings 3.0 Å ligand distance and 2.8 Å method distance used in the present work overlayed with all docked water molecules (transparent) from **A**). **D**) Crystallographic water (*wet*) shown as non-bonded spheres in 6 Å environment of ligand overlayed with water molecules from **C**).

4. References

(1) Yang, X.-L.; Otero, F. J.; Skene, R. J.; McRee, D. E.; Schimmel, P.; Ribas de Pouplana, L. Crystal Structures That Suggest Late Development of Genetic Code Components for Differentiating Aromatic Side Chains. *Proceedings of the National Academy of Sciences* **2003**, *100* (26), 15376–15380. https://doi.org/10.1073/pnas.2136794100.

(2) Card, G. L.; Blasdel, L.; England, B. P.; Zhang, C.; Suzuki, Y.; Gillette, S.; Fong, D.; Ibrahim, P. N.; Artis, D. R.; Bollag, G.; Milburn, M. V; Kim, S.-H.; Schlessinger, J.; Zhang, K. Y. J. A Family of Phosphodiesterase Inhibitors Discovered by Cocrystallography and Scaffold-Based Drug Design. *Nat Biotechnol* **2005**, *23* (2), 201–207. https://doi.org/10.1038/nbt1059.

(3) Johnson, C. N.; Erlanson, D. A.; Murray, C. W.; Rees, D. C. Fragment-to-Lead Medicinal Chemistry Publications in 2015. *Journal of Medicinal Chemistry*. American Chemical Society January 12, 2017, pp 89–99. https://doi.org/10.1021/acs.jmedchem.6b01123.

(4) Johnson, C. N.; Erlanson, D. A.; Jahnke, W.; Mortenson, P. N.; Rees, D. C. Fragment-to-Lead Medicinal Chemistry Publications in 2016. *J Med Chem* **2018**, *61* (5), 1774–1784. https://doi.org/10.1021/acs.jmedchem.7b01298.

(5) Mortenson, P. N.; Erlanson, D. A.; de Esch, I. J. P.; Jahnke, W.; Johnson, C. N. Fragment-to-Lead Medicinal Chemistry Publications in 2017. *J Med Chem* **2019**, *62* (8), 3857–3872. https://doi.org/10.1021/acs.jmedchem.8b01472.

(6) Erlanson, D. A.; de Esch, I. J. P.; Jahnke, W.; Johnson, C. N.; Mortenson, P. N. Fragment-to-Lead Medicinal Chemistry Publications in 2018. *J Med Chem* **2020**, *63* (9), 4430–4444. https://doi.org/10.1021/acs.jmedchem.9b01581.

(7) Jahnke, W.; Erlanson, D. A.; de Esch, I. J. P.; Johnson, C. N.; Mortenson, P. N.; Ochi, Y.; Urushima, T. Fragment-to-Lead Medicinal Chemistry Publications in 2019. *J Med Chem* **2020**, *63* (24), 15494–15507. https://doi.org/10.1021/acs.jmedchem.0c01608.

(8) de Esch, I. J. P.; Erlanson, D. A.; Jahnke, W.; Johnson, C. N.; Walsh, L. Fragment-to-Lead Medicinal Chemistry Publications in 2020. *J Med Chem* **2022**, *65* (1), 84–99. https://doi.org/10.1021/acs.jmedchem.1c01803.
